# Integrative analysis of DNA methylation and inflammatory protein biomarkers in hypertension

**DOI:** 10.3389/fimmu.2026.1671540

**Published:** 2026-02-11

**Authors:** Min Zhu, Jin Zhang, Jing Ma, Xiaofeng Tang, Yan Wang, Dingliang Zhu

**Affiliations:** Department of Cardiovascular Medicine, Research Center for Hypertension Management and Prevention in Community, Shanghai Key Laboratory of Hypertension, Shanghai Institute of Hypertension, State Key Laboratory of Medical Genomics, Ruijin Hospital, Shanghai Jiaotong University School of Medicine, Shanghai, China

**Keywords:** DNA methylation, epigenome-wide analysis, hypertension, inflammatory biomarkers, target organ damage

## Abstract

**Background and objective:**

To investigate the relationship between inflammation and hypertension by comparing epigenetic and serum biomarkers of inflammation and their association with target organ damage (TOD).

**Methods:**

The epigenome-wide methylation profiles of peripheral leukocyte DNA from 176 patients with hypertension were analyzed using Illumina Infinium Methylation EPIC BeadChips. The commercially available Olink^®^ Target 96 Inflammation panels were utilized to evaluate markers associated with inflammation. We identified CpG-protein association protein quantitative trait methylation loci (pQTMs) using mixed linear regression, adjusting for potential confounders. The possible roles of the discovered pQTMs were ascertained by Kyoto Encyclopedia of Genes and Genomes (KEGG) and Gene Ontology (GO) pathway analyses. The association between pQTMs and TOD was also analyzed.

**Results:**

In our analysis, we found 771 significant associations across 11 biomarkers, with a false discovery rate (FDR) of less than 0.05. Lambda estimates ranged from 0.847 to 1.202. Among these, 39 pQTMs showed an association with urine albumin-creatinine ratio, while 82 pQTMs showed an association with carotid intima-media thickness.

**Conclusion:**

Our findings contribute to the understanding of inflammatory biomarkers associated with alterations in DNA methylation in hypertension. However, these results will need to be validated in future studies.

## Introduction

Hypertension ranks as the leading cause of cardiovascular disease and premature death, affecting over one billion adults globally (1). It is thought to be responsible for roughly 54% of strokes and 47% of ischemic heart diseases (2). A number of diseases are largely caused by low-grade inflammation. There is growing evidence that the immune system and an activated inflammatory response play crucial roles in the development of hypertension and are linked to its complications, including myocardial infarction, hemorrhagic stroke, and renal damage (3). It has been demonstrated that the cytokines IL-17, IFN-γ, TNFα, and IL-6 can cause damage to the blood vessels and kidneys, as well as increase sodium retention and systemic vascular resistance (4).

Cytosine-guanine (CpG) dinucleotides are among the most extensively profiled blood-based epigenetic modifications on a large scale. Their methylation (DNAm) can result from exposure to risk factors, such as obesity (5) or smoking (6), impacting regulatory regions of the genome. Additionally, diseases like cancer can lead to global changes in CpG methylation (7). Methylome-wide association studies of protein levels can quantify associations between DNAm at CpGs and protein levels, which are referred to as protein quantitative trait methylation loci (pQTMs) (8). Epigenome-wide association studies (EWAS) have been conducted by several researches on the expression levels of a select group of inflammatory proteins, such as interleukins, IFN-γ, TGF-β, and TNF (9, 10). A meta-analysis of EWAS of CRP revealed multiple inflammation-related CpG sites linked to cardiometabolic phenotypes and coronary heart disease (11). Recently, several studies have focused on the combined effects of multi-omics on clinical phenotypes. Zaghlool et al. performed an EWAS of 1,123 proteins, which identified networks of chronic low-grade inflammatory biomarkers that overlapped with transcriptomics, metabolomics, and clinical endpoints (12). Hillary et al. conducted EWAS on 92 neurological proteins and identified 26 methylation sites associated with nine proteins related to neurological, immunological, and extracellular matrix metabolic pathways (13).

**Figure 1:**
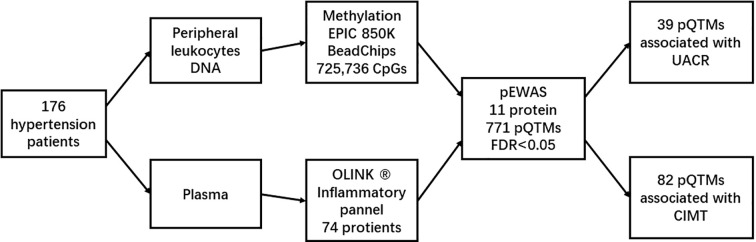
Study design.

However, research focusing on the influence of inflammatory proteins on changes in DNA methylation patterns in patients with hypertension is scarce. To identify the pathogenic mechanisms underlying inflammation in hypertension, we examined the relationship between serum cytokines detected via Olink Target 96 Inflammation panels and DNA methylation patterns in the peripheral leukocytes of patients with hypertension. Furthermore, we explored their effects on target organ damage (TOD) in hypertension (**Figure 1**).

## Materials and methods

### Study population

The participants in the present study were outpatients with hypertension who were identified in Xinzhuang Community Hospital between May 2012 and December 2015. Of the 176 enrolled patients with hypertension, 110 were males. The average age was 66.2 years, ranging from 50–80 years. Participants were included if they had hypertension, defined as BP ≥ 140/90 mmHg, or were taking antihypertensive medications. Those with previous cardiovascular diseases, including stroke, myocardial infarction, heart failure, or dementia, were excluded from the study. Trained clinicians conducted clinical interviews to gather information on the participants’ history of current smoking, drinking, hypertension, diabetes, and recent drug prescriptions, all within a two-week period. General physical examinations, which included assessments of body height and weight, were conducted by trained nurses. Patients were asked to fast and remove shoes or coats during these examinations. The details of clinical information collection have been described previously (14).

All participants provided written informed consent, and the study protocol was approved by the Ruijin Hospital ethics committee.

### Genome-wide DNA methylation profiling analysis

DNA was isolated from peripheral leukocytes using a DNeasy Blood and Tissue Kit (Qiagen, Hilden, Germany). The purity and concentration of DNA were estimated using a Nanodrop 2000 spectrophotometer (Thermo). Subsequently, 500 ng of DNA was bisulfite-converted using EZ DNA Methylation Kits (Zymo Research, USA).

Genome-wide DNA methylation profiling analysis was performed using Illumina Infinium Methylation EPIC 850 K BeadChips (Illumina, San Diego, CA, USA). The data were mainly analyzed using the lmer package in R. Probes were filtered out if they failed to hybridize with more than three beads in at least 5% of the samples, or if the detection p-value was > 0.01. Additionally, non-CpG probes, SNP-related probes, multi-hit probes, and probes located on the X and Y chromosomes were excluded. Ultimately, 725,736 probes were retained for subsequent analysis. β value (ranging from 0 to 1) was used to represent the methylation level at CpG sites, according to the formula β = *m*/(*m* + *u* + 100), where *m* and *u* represent methylated and unmethylated probe intensities, respectively). Subsequently, the β value was normalized using BMIQ.

### Proteomic analysis

Olink^®^ Target 96 Inflammation panels were used to measure plasma inflammatory markers (Olink, Uppsala, Sweden). These panels analyze 92 biomarkers associated with inflammation using a highly sensitive and specific proximity extension assay technology (12). In short, each target protein is matched with a unique complementary DNA barcode and recognized by double antibodies. The Biomark HD (Fluidigm, South San Francisco, CA, USA) is a high-throughput microfluidic real-time PCR device that is used to quantify these barcodes. The resulting cycle threshold (Ct) data were processed for quality control and normalization using internal and external controls. The final assay read-out was given as the normalized protein expression values, which were expressed in arbitrary log2-scale units and corresponded to higher protein levels.

### TOD estimation

Serum creatinine levels were measured at the central laboratory of Ruijin Hospital using fasting venous blood. The Modification of Diet in Renal Disease equation was used to determine the estimated glomerular filtration rate (eGFR). Urine chemistry tests were also conducted the same day, and one spot urine specimen was collected in the morning. A Siemens ADVIA 2400 automated chemistry analyzer (Siemens Healthcare Diagnostics, USA) and commercially available kits (Shanghai Kehua Bio-Engineering, China) were used to measure the urine albumin-creatinine ratio (UACR).

Following standard protocols, echocardiographic data were obtained using a commercially available device (E9; GE Healthcare, Milwaukee, WI, USA). LV diameters and septal and posterior wall thicknesses were measured in accordance with the guidelines of the American Society of Echocardiography using 2-dimensionally guided M-mode tracings, with a recording speed of 50–100 cm/s. LV mass was calculated from M-mode echocardiograms using the formula described by Devereux et al. The LV mass index (LVMI) in g/m^2^ was indexed from the LV mass to the body surface area.

The intima-media thickness (IMT) of the left and right common carotid arteries was measured using high-resolution ultrasound (E9; GE Healthcare, Milwaukee, WI, USA). The distance between the leading edge of the internal lumen and the media adventitia echo was defined as the IMT. Using the larger IMT measurement found in the bilateral common carotid artery, the final IMT value was calculated.

After the participant had rested in the supine position for at least five minutes, Cf-PWV was measured. Using a validated device (SphygmoCor XCEL; AtCor Medical Pty Ltd, Sydney, Australia), the time delay between the right femoral artery and the right common carotid artery’s rapid upstroke was measured. Using a tape measure, the distance between two recording sites on the body’s surface was calculated to estimate the pulse wave’s travel distance. The distance in meters divided by the time delay in seconds was automatically used to compute Cf-PWV.

Utilizing the Mini-Mental State Examination (MMSE), we conducted an oral examination and evaluated cognitive functioning.

### Statistical analysis

To identify CpG-protein associations (pQTMs), a mixed linear regression as conducted in R (version 4.0.0), adjusting for age, sex, smoking, slide, array, and estimated cell counts. Cellular heterogeneity due to variations in cell population proportions in whole blood (15) was controlled using cell proportion estimates based on the Houseman algorithm (16). Estimated cell counts were generated for B-lymphocytes, CD4^+^ and CD8^+^ T-lymphocytes, natural killer cells, granulocytes, and monocytes using the “estimate cell counts” function in the R package minfi (17, 18). The regression coefficient was estimated using the REML fitting algorithm, and the p-value was estimated using the Satterthwaite method. The association of CpG/protein with the TOD of hypertension (LVMI, IMT, Cf-PWV, UACR, eGFR, and MMSE score) was estimated via linear regression, adjusting for age, sex, and mean arterial BP. To control for multiple testing, we applied false discovery rate (FDR) correction using the Benjamini-Hochberg method.

## Results

### Key clinical parameters in patients with hypertension

The average BP was 137.2/71.4 mmHg. The average values for LVMI, IMT, Cf-PWV, UACR, eGFR, and MMSE score were 101.9 g/m^2^, 0.8 mm, 9.4 m/s, 25.3 mg/g, 99.3 ml/min/1.73 m^2^, and 27.8, respectively (**Supplementary Table 1**).

### EWAS of inflammatory biomarker levels

**Figure 2:**
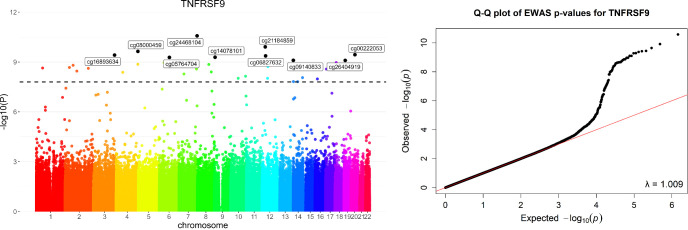
Manhattan plot from the epigenome-wide association studie in 176 patients with hypertension. 725,736 CpG sites were tested for association with the level of TNFRSF9. The black dotted horizontal line represents the FDR< 0.05. QQ plot showing observed vs. expected − log10 (P values) for association at all CpG sites.

In total, 74 of the 92 biomarkers passed the quality control and were used in the analysis. Through EWAS, we identified 771 significant associations across the abundance levels of 11 biomarkers (FDR<0.05). The lambda estimates for these associations ranged from 0.847 to 1.202 (**Figure 2**, **Supplementary Figure 1**). Among these associations, 557 pQTMs were annotated to known genes. Specifically, 74 werelocated within the TSS200 region, 81 within TSS1500, and 74 within the 5’UTR (**Supplementary Table 2**). Notably, the methylation levels at 197 loci showed a positive association with protein abundance, while 360 associations were negative. Twenty regions contained multiple biomarker-associated CpG sites, including those associated with FGF5, FGF23, NTF3, IL10RA, FGF5, and CD274. The degree of methylation of the 13 genes was related to multiple proteins, whereas the level of methylation at loci within PTPRN2 was associated with the abundance of CCL7, FGF23, and FGF5. The top, differentially methylated positions associated with each biomarker in patients with hypertension are shown in **Table 1**.

**Table 1:** Top 11 differentially methylated positions associated with each biomarker in patients with hypertension.

Protein	Probe ID	Estimate	Std. Error	FDR	CHR	RefGene	Region	CpG_Island
CCL7	cg23830969	-10.48	1.17	2.22E-08	3	EAF1	Body	
CD244	cg07240937	11.05	1.67	2.69E-02	1	NPR1	TSS200	Island
CD274	cg02892402	-8.8	1.17	1.53E-04	16	RGS11	Body;	S_Shore
CXCL9	cg12132297	-9.35	1.26	2.74E-04	12	BLOC1S1	TSS1500	S_Shelf
FGF5	cg16987765	-3.01	0.3	1.09E-10	7			
FGF23	cg02095624	-6	0.64	1.77E-09	10	AKR1C8P	Body	
IL6	cg26049527	-5.61	0.78	1.43E-03	1	LHX4	Body	
IL10RA	cg08685054	-11.42	1.26	2.00E-08	8			
NTF3	cg21913681	2.59	0.37	2.02E-03	12	TBX3	TSS200;	Island
TNFRSF9	cg24468104	-5.71	0.8	1.43E-03	7	LINC01006	Body	
PLAU	cg17168630	-3.77	0.42	3.45E-08	19	PLAUR	TSS1500	S_Shore

### pQTMs and TOD in hypertension

**Figure 3:**
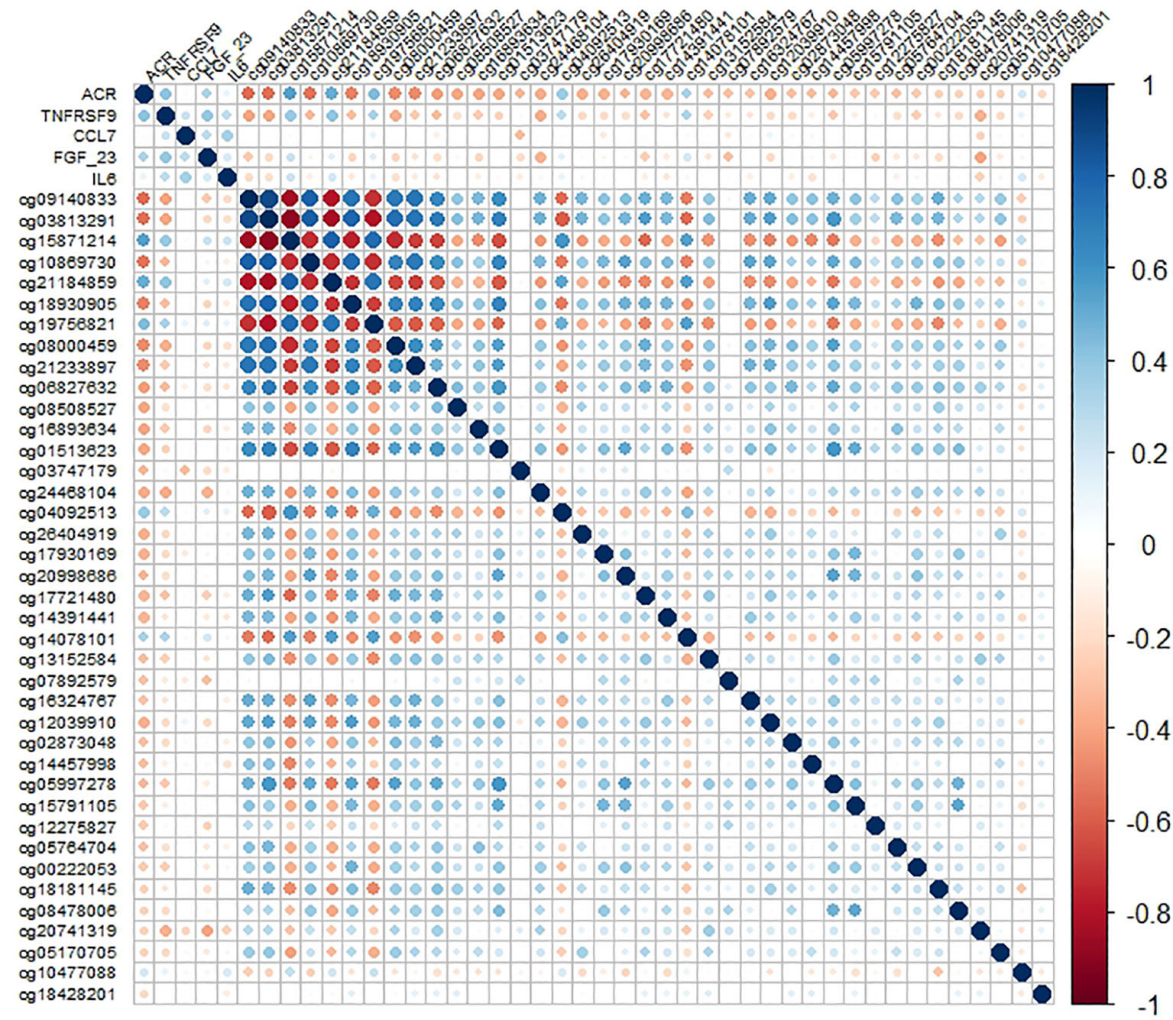
Pairwise correlation matrix for 4 proteins abundance with 39 methylation level of pQTMs assocated with ACR.

Considering that inflammatory factors are involved in the damage process of target organs in hypertension, we further evaluated the association of the 771 pQTMs with LVMI, IMT, Cf-PWV, UACR, eGFR, and MMSE scores using Pearson’s correlation and multiple linear regression analysis. Thirty-nine pQTMs were associated with UACR, even after adjusting for risk factors, including age, sex, BMI, current smoking, SBP, and LDL (**Table 2**). These pQTMs corresponded to four proteins: TNFRSF9, CCL7, FGF23, and IL6, and the expression levels of these five proteins were positively correlated (**Figure 3**). The methylation levels of most loci were negatively correlated with the abundance of fourproteins, except cg04092513, cg10477088, cg10478101, cg15871214, cg19756821, and cg21184859. Of the 39 pQTMs, 33 were related to TNFRSR9. Additionally, 82 pQTMs were found to be associated with CIMT after adjusting for risk factors. Among these, the methylation levels of 59 CpG sites were negatively correlated with protein abundance. These 82 pQTMs encompassed three unique proteins, with NTF3 being associated with 77 CpG sites (**Supplementary Table 3**, **Supplementary Figure 2**). No pQTMs were associated with LVMI, Cf-PWV, eGFR, or MMSE scores.

**Table 2:** Association of the methylation levels of pQTMs with ACR.

	β	FDR	Related protein in present study	Related protein in other study
cg09140833	-2.74	7.32E-17	TNFRSF9	
cg03813291	-3.37	1.23E-16	TNFRSF9	
cg15871214	3.68	2.02E-15	TNFRSF9	IGSF3
cg10869730	-2.5	2.39E-15	TNFRSF9	
cg21184859	4.22	3.03E-13	TNFRSF9	
cg18930905	-4.71	1.31E-12	TNFRSF9	
cg19756821	7.52	4.45E-11	TNFRSF9	
cg08000459	-4	5.18E-11	TNFRSF9	
cg21233897	-3.54	8.94E-11	TNFRSF9	
cg06827632	-5.19	1.06E-08	TNFRSF9	
cg08508527	-10.48	1.73E-08	TNFRSF9	
cg16893634	-12.22	3.80E-08	TNFRSF9	
cg01513623	-2.73	6.45E-08	TNFRSF9	
cg03747179	-12.51	9.09E-07	CCL7	
cg24468104	-11.44	1.93E-06	TNFRSF9	
cg04092513	10.14	3.10E-06	TNFRSF9	
cg26404919	-10.46	3.30E-06	TNFRSF9	F11
cg17930169	-6.34	4.55E-06	TNFRSF9	
cg20998686	-5.92	4.78E-06	TNFRSF9	
cg17721480	-5.71	1.55E-05	TNFRSF9	
cg14391441	-8.78	2.27E-05	TNFRSF9	
cg14078101	4.39	2.71E-05	TNFRSF9	
cg13152584	-6.94	3.79E-05	TNFRSF9	
cg07892579	-2.34	5.09E-05	FGF23	
cg16324767	-7.77	6.97E-05	TNFRSF9	
cg12039910	-8.82	8.60E-05	TNFRSF9	
cg02873048	-8.35	1.15E-04	TNFRSF9	
cg14457998	-8.15	1.25E-04	TNFRSF9	
cg05997278	-4.24	2.36E-04	TNFRSF9	
cg15791105	-2.49	3.40E-04	TNFRSF9	
cg12275827	-5.63	3.77E-04	FGF23	
cg05764704	-11.4	8.34E-04	TNFRSF9	
cg00222053	-6.53	9.23E-04	TNFRSF9	
cg18181145	-10.25	2.35E-03	TNFRSF9	
cg08478006	-2.68	3.27E-03	TNFRSF9	
cg20741319	-2.02	4.46E-03	FGF23	
cg05170705	-3.06	6.12E-03	TNFRSF9	
cg10477088	9.22	7.71E-03	IL6	
cg18428201	-2.46	1.05E-02	TNFRSF9	

β: regression coefficient, using multiple linear regression analysis. Adjusted for age, sex, BMI, smoking, SBP, LDL and glucose.

### Functional enrichment analysis

**Figure 4:**
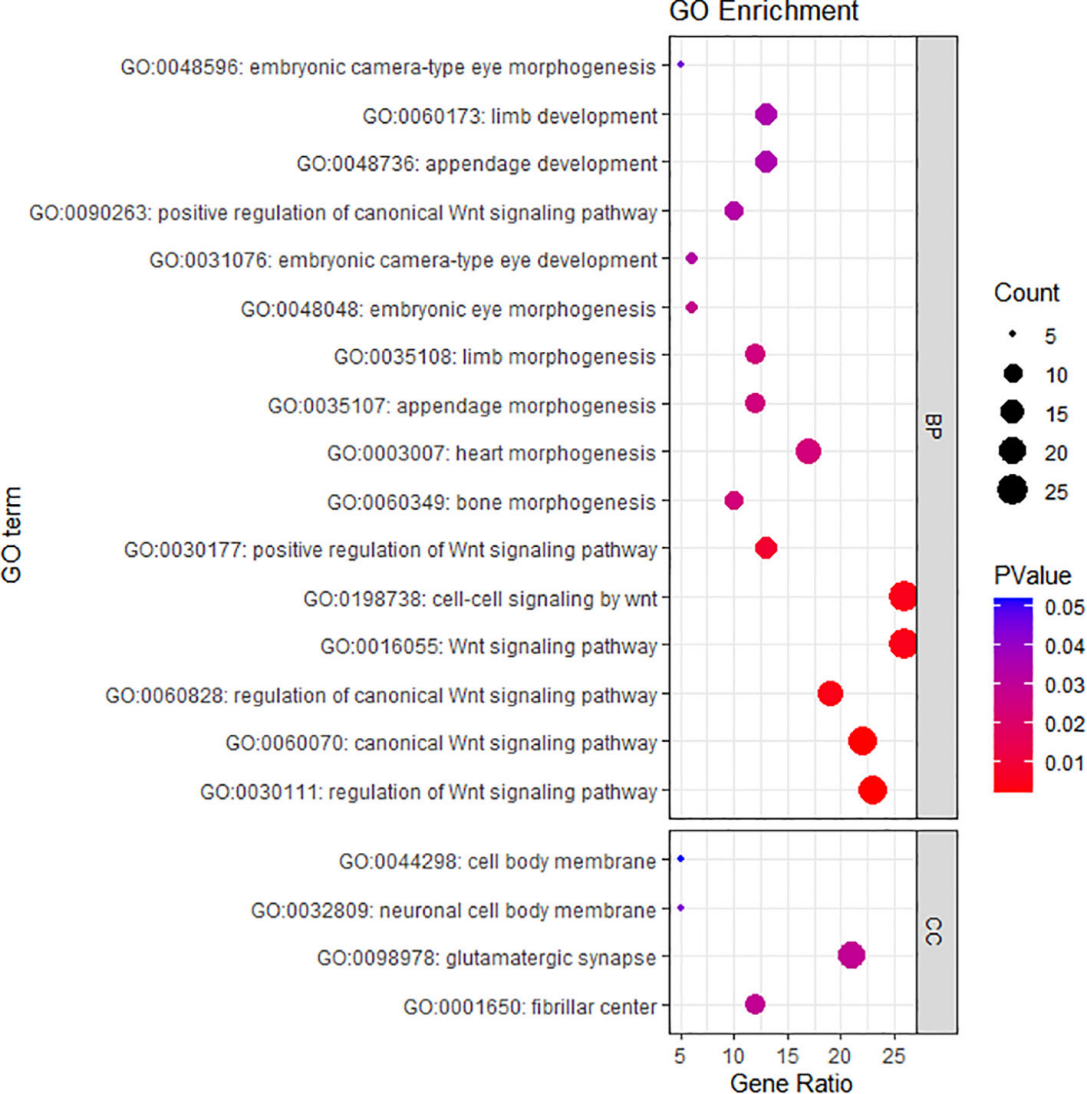
GO pathway enrichment analysis was conducted for the 771 DMPs correlated with inflammatory biomarkers using the R package clusterProfiler. The enrichment analysis was performed with a false discovery rate (FDR) < 0.05 as the threshold for statistical significance. The figure displays the top enriched terms in biological process (BP) and cellular component (CC). The size of each bubble represents the number of DMPs enriched in the corresponding term, and the color gradient indicates the FDR value (from blue to red, reflecting increasing enrichment significance).

Gene Ontology (GO) function and Kyoto Encyclopedia of Genes and Genomes (KEGG) pathway enrichment analyses of the top 771 DMPs correlating with inflammatory factors were performed using the R package clusterProfiler. Under the condition of FDR less than 0.05, the differentially methylated genes were found to be involved in two biological processes: organ development and morphogenesis, as well as the Wnt signaling pathway. Regarding cellular components, enriched terms included the cell body membrane, fibrillar center, glutamatergic synapses, and neuronal cell body membrane (**Figure 4**). However, no item was found to be enriched in the KEGG pathway analysis with a FDR of less than 0.05.

## Discussion

In the present study, we performed the first epigenome-wide analysis of DNA methylation changes associated with the levels of multiple inflammatory proteins in patients with hypertension. Our analysis revealed a total of 771 significant associations with the abundance levels of 11 biomarkers. Notably, among these associations, 39 pQTMs were found to be associated with UACR, while 82 pQTMs showed associations with CIMT. Most associations between DNA methylation and protein levels are related to age (19), blood cell composition (20), and rheumatoid arthritis (21). Additionally, many CpG loci that appear in pQTMs are associated with other plasma proteins, including IGSF3, F11, and PGAM1 (8).

Aberrant inflammatory responses result in tissue damage and are central to the pathogenesis of multiple diseases, including diabetes (22), cardiovascular disorders (23), and cognitive dysfunction (24). The chemokines involved in the pathogenesis of hypertension include monocyte chemoattractant protein-1, MCP-1, CCL2, interferon-inducible protein, interleukin-8, RANTES, fractalkine, along with their receptors. However, the mechanisms involving chemokines and their receptors in hypertension are intricate and not fully elucidated (25). Association studies between DNA methylation and chronic low-grade inflammation have primarily focused on scattered proteins, with CRP being a common target. Wielscher et al. (26) carried out a multi-ethnic EWAS to characterize the DNA methylation signature associated with chronic low-grade inflammation, as measured by CRP. They identified 1,511 independent differentially methylated loci associated with CRP levels. Importantly, they found that an activated CpG signature significantly increased the risk of cardiometabolic diseases and COPD. However, few studies have detected an association between genome-wide methylation alterations and multiple inflammatory proteins, particularly in patients with hypertension. Hillary et al. (27) performed an EWAS on the levels of 70 plasma-derived inflammatory protein biomarkers in healthy, older adults. They identified three CpG sites spread across three proteins (CCL11, IL18R1, and CXCL9) and demonstrated putative causal relationships between IL18R1 and inflammatory bowel disease. No overlap was observed between our results and those reported by Hillary et al. Such inconsistencies may be induced by different disease backgrounds that influence the interaction between immune factors and DNA methylation.

Most of the 39 pQTMs related to UACR were linked to TNFRSF9, with 34 out of 39 being specifically related to this protein. TNFRSF9, also known as CD137 or 4-1BB, belongs to a group of costimulatory immune receptors and is a member of the tumor necrosis factor receptor (TNF-R) superfamily. It is preferentially found on activated T cells, regulatory T cells (Tregs), and innate immune cells (28). TNFRSF9 is implicated in various immunomodulatory responses related to kidney disease, as well as biological processes like cell survival, proliferation, and death. In a study by Nano et al. (29), TNFRSF9 was associated with baseline UACR and an increased risk of incident chronic kidney disease, independent of other cardiorenal risk factors. A similar study by Niewczas et al. found a kidney risk inflammatory signature consisting of 17 inflammatory proteins enriched for TNF-R superfamily members in participants from three different cohorts with type 1 and type 2 diabetes (30), which significantly increased the 10-year risk of end-stage renal disease. The level of TNFRSF9 was positively related to UACR (β = 0.4, p = 3.73e-08), consistent with the previous findings. Among the 34 CpGs associated with TNFRSF9, 25 loci were annotated as known genes, wherein fat atypical cadherin 1 (FAT1) was associated with UACR levels in genome-wide association studies (31) and family studies (32). FAT1 is a calcium ion-dependent adhesion protein that belongs to the fat family. Notably, FAT1 regulates cell migration and growth through specific protein–protein interactions in its cytoplasmic tail (33). Moreover, FAT1 is abundantly expressed in the glomerular podocytes; specific deletion of FAT1 in mouse podocytes can induce abnormal development of the glomerular filtration barrier, reduce cell adhesion and migration of fibroblasts and podocytes, and lead to the disappearance of foot processes and slits (31). Renal biopsies of patients with membranous nephropathy after hematopoietic stem cell transplantation also revealed granular staining for FAT1 along the glomerular basement membrane (34). In a study on the pan-cancer landscape of T-cell heterogeneity, Zheng et al. reported that FAT1 mutations were positively correlated with TNFRSF9^+^ Treg cell frequencies. However, the influence of TNFRSF9 on the methylation of FAT1 and its combined effect on UACR requires further study.

Our study has several strengths and limitations. To the best of our knowledge, this is the first study to investigate the association between peripheral blood leukocyte DNA methylation and the levels of multiple inflammatory proteins in patients with hypertension using an 850k chip and an Olink inflammation panel. These results should also be interpreted with consideration of their limitations. First, owing to the relatively small sample size, the risk of false-positive results should be considered, necessitating further replication studies with larger sample sizes. However, to mitigate the impact of this limitation, we applied the Benjamini–Hochberg method to control for FDR. Second, given the descriptive nature of the study, it cannot establish a definitive cause-and-effect relationship. Additional prospective studies are therefore required to confirm our findings. Third, our study included patients with hypertension, which may hinder the applicability of our conclusions to the general population. Nevertheless, previous research has indicated that the disease background can influence methylation changes induced by inflammatory proteins (35, 36). Therefore, targeting DNA methylation changes related to inflammatory protein levels, especially in hypertension, may reveal specific underlying mechanisms. Additionally, we found that pQTMs in hypertension were also related to UACR and CIMT, the surrogate markers of TOD, which offered insights into the underlying function.

In conclusion, we integrated DNA methylation data and inflammatory protein abundance in patients with hypertension and identified 771 novel pQTMs, some of which were related to UACR and CIMT. Our findings contribute to the understanding of the inflammatory factors associated with DNA methylation alterations in hypertension. However, our results will need to be validated in future studies.

## Data Availability

The data presented in the study are deposited in NCBI's Gene Expression Omnibus (GEO) repository, accession number GSE314180 (https://www.ncbi.nlm.nih.gov/geo/query/acc.cgi?acc=GSE314180).
