# Supplementary figures and images for: Integrative analysis of DNA methylation and inflammatory protein biomarkers in hypertension
Source: Front Immunol. 2026 Feb 11;17:1671540. doi: 10.3389/fimmu.2026.1671540 (PMC12932573; doi:10.3389/fimmu.2026.1671540)

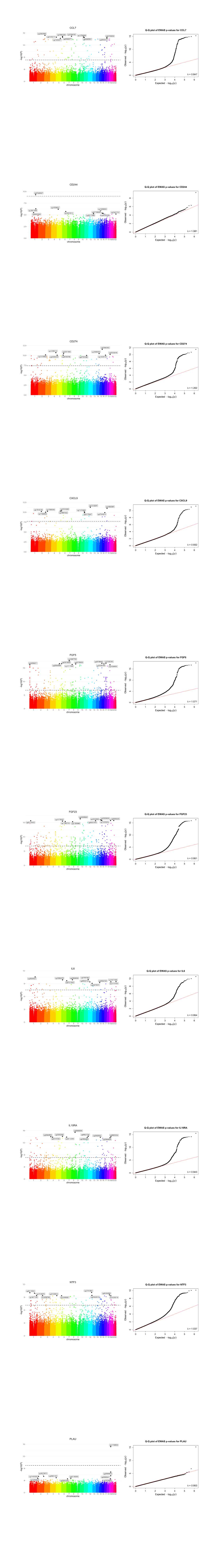

Supplement: Supplementary file 1 [file Image1.tif]

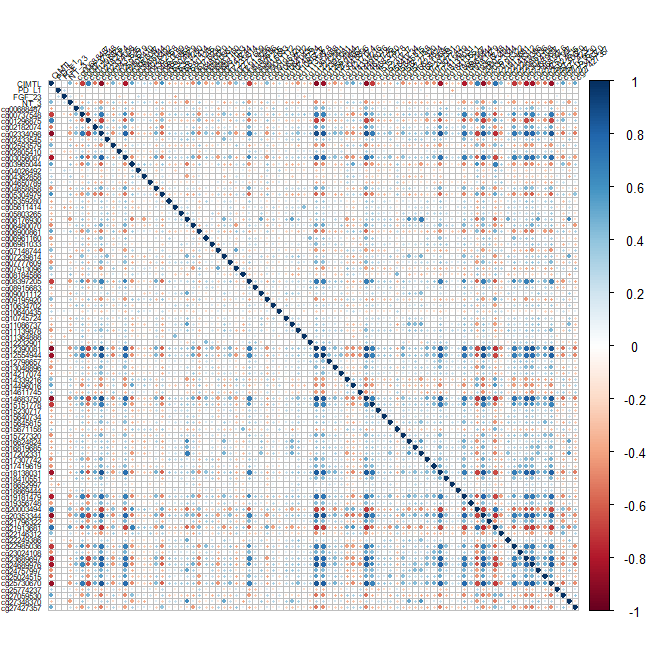

Supplement: Supplementary file 2 [file Image2.tiff]
