# Supplementary material for: Integrative analysis of DNA methylation and inflammatory protein biomarkers in hypertension
Source: Front Immunol. 2026 Feb 11;17:1671540. doi: 10.3389/fimmu.2026.1671540 (PMC12932573; doi:10.3389/fimmu.2026.1671540)
Supplement: Supplementary file 3 [file Table1.docx]

| Characteristics | Value |
| --- | --- |
| Male (n, %) | 110（62.5） |
| Age (years) | 66.2±5.2 |
| Body-mass index (kg/m^2^ ) | 25.0±3.1 |
| Systolic blood pressure (mmHg) | 137.2±16.1 |
| Diastolic blood pressure (mmHg) | 71.4±10.1 |
| Pulse ratio (beats/min) | 74.7±12.5 |
| Low-density lipoprotein cholesterol (mmol/l) | 2.9±0.8 |
| Fasting plasma glucose (mmol/l) | 4.9 (4.6-5.6) |
| Estimated glomerular filtration rate (ml/min/1.73 m2 ) | 99.3±21.3 |
| Albumin-to-creatinine ratio (mg/g) | 25.3 (14.2-43.4) |
| Carotid-femoral Pulse Wave Velocity (m/s) | 9.1 (8.1-10.3) |
| Carotid intima-media thickness (mm) | 0.74(0.67-0.82) |
| Left ventricular mass index (g/m2 ) | 95.2(81.6-117. 2) |
| Mini-mental state examination score | 28(27-29) |

Table 1. Characteristics of study population

Data are presented as mean±SD or median (Q1–Q3), for data not distributed normally.
