# Supplementary material for: Integrative analysis of DNA methylation and inflammatory protein biomarkers in hypertension
Source: Front Immunol. 2026 Feb 11;17:1671540. doi: 10.3389/fimmu.2026.1671540 (PMC12932573; doi:10.3389/fimmu.2026.1671540)
Supplement: Supplementary file 4 [file Table2.docx]

SUPPLEMENTARY TABLE 2. Differentially methylated positions associated with inflammatory factor levels in patients with hypertension

| Protein | Probe ID | Estimate | Std. Error | FDR | CHR | RefGene | Region | CpG_Island |
| --- | --- | --- | --- | --- | --- | --- | --- | --- |
| CCL7 | cg23830969 | -10.48 | 1.17 | 2.22E-08 | 3 | EAF1 | Body |  |
| CCL7 | cg22384183 | -8.88 | 1 | 2.22E-08 | 8 | ARHGEF10 | Body | N_Shelf |
| CCL7 | cg17851230 | -4.15 | 0.47 | 2.22E-08 | 6 | C6orf10 | Body |  |
| CCL7 | cg13708908 | -4.11 | 0.47 | 2.22E-08 | 10 | MXI1 | 5'UTR |  |
| CCL7 | cg04455214 | -8.63 | 0.98 | 2.22E-08 | 5 |  |  |  |
| CCL7 | cg20842677 | -4.1 | 0.47 | 2.22E-08 | 6 | HLA-DOA | Body | N_Shore |
| CCL7 | cg22065591 | -4.15 | 0.47 | 2.22E-08 | 11 | RHOG | Body |  |
| CCL7 | cg03760856 | -8.38 | 0.96 | 2.42E-08 | 17 | TNRC6C | 5'UTR |  |
| CCL7 | cg08502377 | -7.97 | 0.92 | 2.79E-08 | 17 | SEC14L1 | Body | S_Shore |
| CCL7 | cg11454877 | -8.59 | 1 | 2.79E-08 | 5 | CXXC5 | Body | S_Shore |
| CCL7 | cg02647998 | -7.61 | 0.88 | 2.79E-08 | 3 | TIMP4 | TSS1500 | S_Shore |
| CCL7 | cg04024692 | 43.8 | 5.11 | 2.92E-08 | 11 | C11orf58 | TSS200 | Island |
| CCL7 | cg23706557 | -4.03 | 0.47 | 2.92E-08 | 11 | TMEM218 | TSS1500 | S_Shore |
| CCL7 | cg11311032 | -8.66 | 1.01 | 2.92E-08 | 6 |  |  |  |
| CCL7 | cg10924904 | 4.81 | 0.56 | 3.37E-08 | 19 | TIMM13 | TSS200 | S_Shore |
| CCL7 | cg04444703 | -7.85 | 0.92 | 3.37E-08 | 14 | NUMB | Body |  |
| CCL7 | cg19094872 | -7.46 | 0.88 | 3.71E-08 | 2 | PASK | Body |  |
| CCL7 | cg00986987 | -8.34 | 0.98 | 3.71E-08 | 8 | EXTL3 | Body |  |
| CCL7 | cg27063617 | -8.19 | 0.97 | 3.71E-08 | 2 |  |  |  |
| CCL7 | cg10302002 | -7.7 | 0.91 | 3.71E-08 | 15 |  |  |  |
| CCL7 | cg04981630 | -7.56 | 0.89 | 3.71E-08 | 3 | MGLL | Body; |  |
| CCL7 | cg11848882 | -8.78 | 1.04 | 3.71E-08 | 8 |  |  |  |
| CCL7 | cg18070969 | -7.52 | 0.89 | 3.71E-08 | 12 |  |  |  |
| CCL7 | cg05327509 | -9.09 | 1.08 | 3.71E-08 | 13 | PCCA | Body; |  |
| CCL7 | cg26877648 | 4.62 | 0.55 | 3.78E-08 | 17 | MED24 | TSS200; | Island |
| CCL7 | cg25673474 | -3.99 | 0.47 | 3.82E-08 | 4 |  |  |  |
| CCL7 | cg01785288 | -8.36 | 0.99 | 3.82E-08 | 9 |  |  |  |
| CCL7 | cg11976007 | -7.74 | 0.93 | 4.82E-08 | 19 | PIP5K1C | Body | Island |
| CCL7 | cg22384832 | -7.83 | 0.94 | 4.82E-08 | 10 |  |  |  |
| CCL7 | cg23444868 | -7.64 | 0.93 | 9.30E-08 | 17 |  |  |  |
| CCL7 | cg01147665 | -7.51 | 0.91 | 9.49E-08 | 3 |  |  | N_Shore |
| CCL7 | cg06738700 | -8.66 | 1.05 | 1.03E-07 | 1 | RGS13 | TSS1500 |  |
| CCL7 | cg08614201 | -7.43 | 0.91 | 1.51E-07 | 1 | CD160 | 5'UTR | S_Shore |
| CCL7 | cg02678520 | -4.76 | 0.58 | 1.51E-07 | 6 |  |  |  |
| CCL7 | cg26418099 | -8.76 | 1.1 | 3.91E-07 | 10 |  |  | N_Shelf |
| CCL7 | cg11771262 | -7.45 | 0.94 | 4.76E-07 | 8 | GRHL2 | Body |  |
| CCL7 | cg09096995 | -8.08 | 1.03 | 6.51E-07 | 21 |  |  |  |
| CCL7 | cg03099492 | 18.71 | 2.39 | 8.17E-07 | 10 | CYP26A1 | TSS200 | N_Shore |
| CCL7 | cg11962201 | 28.02 | 3.58 | 8.96E-07 | 20 | TRPC4AP | TSS200; | Island |
| CCL7 | cg07146507 | -8.13 | 1.05 | 1.07E-06 | 15 | SECISBP2L | Body |  |
| CCL7 | cg01111746 | -7.15 | 0.93 | 1.73E-06 | 12 | RARG | 3'UTR; |  |
| CCL7 | cg08128650 | -7.29 | 0.95 | 2.27E-06 | 11 | RELA | Body; | N_Shelf |
| CCL7 | cg27380724 | -22.93 | 3 | 2.31E-06 | 19 | BRSK1 | Body | Island |
| CCL7 | cg05048685 | -7.13 | 0.94 | 3.01E-06 | 8 | TSNARE1 | Body |  |
| CCL7 | cg19099892 | -7.08 | 0.93 | 3.11E-06 | 6 |  |  |  |
| CCL7 | cg04524433 | -7.31 | 0.98 | 6.61E-06 | 16 | TRAPPC2L | TSS1500 | S_Shore |
| CCL7 | cg22699052 | -11.34 | 1.54 | 8.34E-06 | 17 | NXN | Body | Island |
| CCL7 | cg25588935 | -18.87 | 2.57 | 1.07E-05 | 7 | PTPRN2 | Body; |  |
| CCL7 | cg05695274 | -7.03 | 0.98 | 2.17E-05 | 6 | GCNT2 | Body; |  |
| CCL7 | cg17102042 | -6.33 | 0.89 | 3.34E-05 | 7 | PTPRN2 | Body; |  |
| CCL7 | cg04103985 | -21.14 | 2.97 | 3.88E-05 | 16 |  |  |  |
| CCL7 | cg03552384 | -41.59 | 5.88 | 4.54E-05 | 2 |  |  |  |
| CCL7 | cg05901384 | -8.13 | 1.15 | 4.73E-05 | 15 | PLIN1 | Body; | S_Shore |
| CCL7 | cg01379412 | -3.25 | 0.46 | 6.75E-05 | 11 | DIXDC1 | Body | S_Shelf |
| CCL7 | cg27388036 | -18.14 | 2.62 | 8.84E-05 | 4 |  |  |  |
| CCL7 | cg23500656 | -19.26 | 2.8 | 1.16E-04 | 3 | DGKG | Body; |  |
| CCL7 | cg06265809 | -6.89 | 1.02 | 2.25E-04 | 14 | MIR496 | TSS1500; |  |
| CCL7 | cg05103397 | -3.03 | 0.45 | 2.85E-04 | 17 | WSCD1 | Body |  |
| CCL7 | cg22956084 | -5.51 | 0.82 | 3.43E-04 | 8 |  |  |  |
| CCL7 | cg00524064 | -5.72 | 0.86 | 3.73E-04 | 5 | SMIM23 | TSS1500 |  |
| CCL7 | cg02656667 | 28.36 | 4.3 | 5.03E-04 | 6 | HIST1H4E | TSS200 | N_Shore |
| CCL7 | cg27505587 | 21.77 | 3.36 | 9.00E-04 | 20 |  |  | N_Shelf |
| CCL7 | cg19060382 | 37.96 | 5.87 | 9.38E-04 | 12 | LOC253724 | TSS1500 | S_Shore |
| CCL7 | cg25691398 | -6 | 0.93 | 1.01E-03 | 16 |  |  |  |
| CCL7 | cg07074666 | 5.66 | 0.88 | 1.02E-03 | 2 | EFR3B | Body | Island |
| CCL7 | cg03747179 | -5.53 | 0.86 | 1.26E-03 | 16 |  |  |  |
| CCL7 | cg24915371 | 26.65 | 4.17 | 1.33E-03 | 2 | PDE6D | TSS1500 | Island |
| CCL7 | cg02371401 | -24.99 | 3.95 | 1.81E-03 | 5 | TPPP | Body | N_Shore |
| CCL7 | cg19996406 | -5.2 | 0.83 | 2.82E-03 | 8 |  |  |  |
| CCL7 | cg03894103 | -4.9 | 0.79 | 3.34E-03 | 2 | PREPL | 5'UTR | N_Shelf |
| CCL7 | cg21331461 | 3.35 | 0.54 | 4.20E-03 | 12 | KCNJ8 | TSS200 |  |
| CCL7 | cg26392145 | -6.08 | 0.99 | 4.37E-03 | 19 | ZNF599 | Body |  |
| CCL7 | cg14186944 | -9.43 | 1.55 | 5.59E-03 | 9 |  |  |  |
| CCL7 | cg00847504 | -4.07 | 0.67 | 5.93E-03 | 17 | SPNS3 | Body |  |
| CCL7 | cg20846192 | -6.72 | 1.11 | 6.66E-03 | 7 |  |  |  |
| CCL7 | cg20097090 | -4.87 | 0.81 | 8.94E-03 | 7 |  |  | S_Shelf |
| CCL7 | cg26205041 | -5.69 | 0.95 | 9.35E-03 | 5 |  |  |  |
| CCL7 | cg08084084 | -3.08 | 0.52 | 1.15E-02 | 7 | HECW1 | Body; |  |
| CCL7 | cg19764764 | 5.64 | 0.96 | 1.66E-02 | 10 | LINC00838 | TSS1500 |  |
| CCL7 | cg27256177 | -5.23 | 0.89 | 1.80E-02 | 12 | PLEKHA5 | Body; |  |
| CCL7 | cg17276118 | -15.71 | 2.7 | 1.95E-02 | 11 | FAT3 | TSS1500 |  |
| CCL7 | cg21422291 | -4.08 | 0.71 | 2.46E-02 | 1 | LRRC8B | Body; |  |
| CCL7 | cg05222618 | -3.74 | 0.65 | 2.67E-02 | 6 |  |  |  |
| CCL7 | cg06658912 | -2.63 | 0.46 | 2.77E-02 | 6 | ROS1 | TSS1500 |  |
| CCL7 | cg13284705 | -8.4 | 1.47 | 3.10E-02 | 19 | KLK6 | 5'UTR |  |
| CCL7 | cg17207815 | 23.98 | 4.2 | 3.24E-02 | 8 | NUDT18 | Body | N_Shore |
| CCL7 | cg21199251 | -7.9 | 1.4 | 4.92E-02 | 1 | AJAP1 | Body; | N_Shelf |
| CD274 | cg02892402 | -8.8 | 1.17 | 1.53E-04 | 16 | RGS11 | Body; | S_Shore |
| CD274 | cg12396412 | -6.28 | 0.87 | 4.44E-04 | 4 | ARHGAP10 | Body |  |
| CD274 | cg22411864 | -6.72 | 0.94 | 4.44E-04 | 6 | DNAH8 | Body |  |
| CD274 | cg16469463 | -5.73 | 0.81 | 4.44E-04 | 14 | ASPG | Body |  |
| CD274 | cg00239446 | -2.96 | 0.42 | 5.17E-04 | 19 | FKBP8 | Body; |  |
| CD274 | cg13159823 | -3.51 | 0.5 | 5.17E-04 | 2 | HDAC4 | Body | S_Shore |
| CD274 | cg08190786 | -5.97 | 0.86 | 5.17E-04 | 6 |  |  |  |
| CD274 | cg15028103 | -5.96 | 0.86 | 5.17E-04 | 17 | KCNJ12 | TSS1500 | N_Shore |
| CD274 | cg02263444 | -3.48 | 0.5 | 5.17E-04 | 5 |  |  |  |
| CD274 | cg07707380 | -5.57 | 0.81 | 5.30E-04 | 11 | PCNXL3 | Body |  |
| CD274 | cg14387909 | -3.29 | 0.48 | 5.30E-04 | 9 | KCNT1 | Body | S_Shore |
| CD274 | cg13043541 | -2.89 | 0.42 | 5.45E-04 | 1 | CAMTA1 | Body |  |
| CD274 | cg02375938 | -3.87 | 0.56 | 5.45E-04 | 5 | RAPGEF6 | Body; |  |
| CD274 | cg09849532 | -2.88 | 0.42 | 6.23E-04 | 20 | ARFGEF2 | Body |  |
| CD274 | cg04769577 | -5.25 | 0.77 | 6.45E-04 | 7 | GNA12 | TSS200 |  |
| CD274 | cg05790579 | -7.1 | 1.05 | 6.50E-04 | 3 | IP6K1 | 5'UTR | N_Shore |
| CD274 | cg12821702 | -5.57 | 0.82 | 6.94E-04 | 2 |  |  |  |
| CD274 | cg03598185 | -7.7 | 1.14 | 6.94E-04 | 19 | SCAMP4 | Body | S_Shore |
| CD274 | cg12053595 | -5.85 | 0.87 | 6.94E-04 | 20 | ZBTB46 | 3'UTR | N_Shore |
| CD274 | cg09341108 | -5.97 | 0.89 | 7.49E-04 | 14 | CDC42BPB | Body |  |
| CD274 | cg08358625 | -6.02 | 0.9 | 7.49E-04 | 10 | KNDC1 | Body | Island |
| CD274 | cg25027698 | -2.81 | 0.42 | 7.96E-04 | 3 | ACAD11 | Body; |  |
| CD274 | cg18652997 | -3.02 | 0.45 | 7.96E-04 | 4 | SORCS2 | Body |  |
| CD274 | cg22886200 | -2.83 | 0.42 | 8.16E-04 | 15 | SEMA6D | 5'UTR |  |
| CD274 | cg07478893 | -5.9 | 0.89 | 9.27E-04 | 8 | ZFPM2 | Body |  |
| CD274 | cg23527366 | -5.67 | 0.85 | 9.27E-04 | 3 | ABHD14A | Body | S_Shore |
| CD274 | cg19714595 | -8.04 | 1.21 | 9.42E-04 | 1 | ARL8A | Body | N_Shore |
| CD274 | cg17477995 | 3.28 | 0.5 | 1.15E-03 | 1 | ERRFI1 | 5'UTR | Island |
| CD274 | cg14281482 | -4.93 | 0.75 | 1.18E-03 | 17 | AATK | Body | Island |
| CD274 | cg05245515 | -5.37 | 0.82 | 1.44E-03 | 14 | SLC39A2 | TSS200 |  |
| CD274 | cg21669226 | -2.76 | 0.42 | 1.44E-03 | 8 |  |  |  |
| CD274 | cg01955313 | -6.99 | 1.08 | 1.56E-03 | 1 |  |  |  |
| CD274 | cg16015543 | -5.95 | 0.92 | 1.56E-03 | 12 |  |  |  |
| CD274 | cg09796800 | -17.11 | 2.64 | 1.56E-03 | 4 | SLC25A31 | TSS200 | Island |
| CD274 | cg04533487 | 4.8 | 0.75 | 1.94E-03 | 3 | UBA5 | TSS1500 | Island |
| CD274 | cg00041599 | 2.01 | 0.31 | 2.68E-03 | 5 | UNC5A | Body | S_Shore |
| CD274 | cg12582181 | -5.46 | 0.86 | 2.68E-03 | 3 |  |  |  |
| CD274 | cg07485541 | -5.57 | 0.88 | 2.68E-03 | 11 | TMEM216 | 3'UTR |  |
| CD274 | cg17548367 | -6.31 | 0.99 | 2.68E-03 | 3 | ARHGAP31 | Body |  |
| CD274 | cg25290633 | -6.08 | 0.96 | 2.68E-03 | 10 | ABCC2 | Body | S_Shore |
| CD274 | cg03662501 | -5.32 | 0.86 | 6.79E-03 | 20 | TPD52L2 | Body |  |
| CD274 | cg07538339 | 2.95 | 0.48 | 9.33E-03 | 8 | RUNX1T1 | 5'UTR |  |
| CD274 | cg06527989 | 2.31 | 0.38 | 1.23E-02 | 5 | UNC5A | Body | S_Shore |
| CD274 | cg10398905 | 3.26 | 0.54 | 1.23E-02 | 2 | LMAN2L | TSS1500 | S_Shore |
| CD274 | cg20371573 | -4.7 | 0.78 | 1.51E-02 | 13 | MYO16 | Body | S_Shore |
| CD274 | cg12506664 | -5.58 | 0.93 | 1.62E-02 | 5 | KCNIP1 | 5'UTR | S_Shore |
| CD274 | cg18778450 | -9.17 | 1.54 | 1.85E-02 | 7 | LRCH4 | Body; | N_Shelf |
| CD274 | cg12271587 | -4.34 | 0.73 | 1.85E-02 | 9 | TTF1 | TSS1500 | S_Shore |
| CD274 | cg02279953 | -4.84 | 0.81 | 1.98E-02 | 14 | SNORD114-11 | TSS1500 |  |
| CD274 | cg13022387 | -3.74 | 0.64 | 2.44E-02 | 1 |  |  |  |
| CD274 | cg25115503 | -4.36 | 0.75 | 2.82E-02 | 2 |  |  | N_Shelf |
| CD274 | cg15403283 | -2.66 | 0.46 | 3.07E-02 | 14 | SPTB;SPTB | 3'UTR |  |
| CD274 | cg23635546 | -17.64 | 3.04 | 3.33E-02 | 1 |  |  |  |
| CD274 | cg00065215 | 2.97 | 0.52 | 4.24E-02 | 1 | PAX7 | Body; | Island |
| CXCL9 | cg12132297 | -9.35 | 1.26 | 2.74E-04 | 12 | BLOC1S1 | TSS1500 | S_Shelf |
| CXCL9 | cg18563987 | -10.08 | 1.38 | 2.74E-04 | 17 | SAP30BP | Body |  |
| CXCL9 | cg27610206 | -8.92 | 1.26 | 5.54E-04 | 5 |  |  |  |
| CXCL9 | cg19667423 | -10.44 | 1.48 | 5.54E-04 | 5 |  |  |  |
| CXCL9 | cg17985556 | -4.67 | 0.67 | 5.54E-04 | 3 | CADPS | Body; |  |
| CXCL9 | cg17016175 | -10.16 | 1.45 | 5.54E-04 | 2 | TMEM131 | Body |  |
| CXCL9 | cg21373880 | -9.33 | 1.34 | 6.29E-04 | 10 | ATE1 | 5'UTR |  |
| CXCL9 | cg11924452 | -5.47 | 0.79 | 6.36E-04 | 2 |  |  | Island |
| CXCL9 | cg07930510 | -4.92 | 0.71 | 6.78E-04 | 16 | BANP | Body; | N_Shore |
| CXCL9 | cg01170247 | -9.05 | 1.32 | 6.78E-04 | 10 | INPP5A | Body | S_Shore |
| CXCL9 | cg14603991 | -9.96 | 1.47 | 1.00E-03 | 1 |  |  |  |
| CXCL9 | cg00105475 | -8.57 | 1.27 | 1.10E-03 | 2 |  |  |  |
| CXCL9 | cg06921552 | 14.94 | 2.24 | 1.53E-03 | 11 |  |  | Island |
| CXCL9 | cg09042010 | -8.71 | 1.32 | 1.95E-03 | 10 | AIFM2 | Body; |  |
| CXCL9 | cg08563982 | -10.02 | 1.52 | 1.95E-03 | 6 | C6orf10 | Body |  |
| CXCL9 | cg03974777 | -9.48 | 1.44 | 2.01E-03 | 21 | C21orf59 | Body; |  |
| CXCL9 | cg06416938 | -10.7 | 1.65 | 3.26E-03 | 12 | VWF | Body |  |
| CXCL9 | cg09899825 | -4.67 | 0.72 | 3.26E-03 | 3 | TRAK1 | TSS200; |  |
| CXCL9 | cg25624541 | -8.59 | 1.33 | 3.58E-03 | 17 | VPS53 | Body; |  |
| CXCL9 | cg05971321 | -14.31 | 2.23 | 3.89E-03 | 16 | TSC2 | Body; | Island |
| CXCL9 | cg02944999 | -10.87 | 1.73 | 6.50E-03 | 2 | CPS1 | Body; |  |
| CXCL9 | cg01708091 | -4.68 | 0.74 | 6.50E-03 | 6 | SCAND3 | Body | N_Shelf |
| CXCL9 | cg08578765 | -5.32 | 0.85 | 8.22E-03 | 7 | PLXNA4 | Body |  |
| CXCL9 | cg14803762 | -5.05 | 0.81 | 9.01E-03 | 11 | TH | Body; | S_Shelf |
| CXCL9 | cg10016285 | -9.95 | 1.63 | 1.60E-02 | 7 |  |  |  |
| CXCL9 | cg14446017 | -11.56 | 1.9 | 1.75E-02 | 10 | CUBN | Body |  |
| CXCL9 | cg20744894 | -8.37 | 1.38 | 1.91E-02 | 5 |  |  |  |
| CXCL9 | cg21584710 | -4.75 | 0.8 | 2.94E-02 | 17 | DNAI2 | Body |  |
| CXCL9 | cg06735585 | -8 | 1.35 | 3.61E-02 | 1 | ALPL | 5'UTR |  |
| CXCL9 | cg16945514 | -9.32 | 1.58 | 3.61E-02 | 3 | CLRN1 | Body |  |
| CXCL9 | cg05222618 | -5.36 | 0.91 | 3.65E-02 | 6 |  |  |  |
| FGF5 | cg16987765 | -3.01 | 0.3 | 1.09E-10 | 7 |  |  |  |
| FGF5 | cg23392383 | -2.83 | 0.29 | 2.50E-10 | 17 | KIF18B | 5'UTR |  |
| FGF5 | cg05796992 | 6.39 | 0.67 | 2.50E-10 | 15 | LRRK1 | Body | N_Shore |
| FGF5 | cg03918629 | -6.25 | 0.66 | 2.50E-10 | 6 | MYLK4 | Body |  |
| FGF5 | cg25604984 | 5.29 | 0.56 | 2.50E-10 | 5 | YTHDC2 | TSS200 | N_Shore |
| FGF5 | cg25399166 | -2.96 | 0.31 | 2.50E-10 | 16 | FAM65A | Body | S_Shelf |
| FGF5 | cg20749005 | -6.02 | 0.63 | 2.50E-10 | 8 | TRAPPC9 | Body |  |
| FGF5 | cg12117590 | -3.12 | 0.33 | 3.16E-10 | 8 |  |  |  |
| FGF5 | cg24094804 | -2.83 | 0.3 | 3.16E-10 | 19 | LPAR2 | Body | Island |
| FGF5 | cg12050271 | -5.65 | 0.6 | 3.16E-10 | 1 | FLJ42875 | Body | Island |
| FGF5 | cg20516256 | -6.14 | 0.66 | 3.16E-10 | 14 | SFRS5 | TSS1500 | N_Shore |
| FGF5 | cg08489279 | -5.76 | 0.62 | 3.16E-10 | 9 |  |  |  |
| FGF5 | cg09869811 | -5.48 | 0.59 | 3.35E-10 | 16 | NUTF2 | 5'UTR |  |
| FGF5 | cg12511236 | -6.2 | 0.67 | 3.35E-10 | 15 |  |  |  |
| FGF5 | cg24538089 | 5.07 | 0.55 | 4.37E-10 | 15 | LRRK1 | Body | N_Shore |
| FGF5 | cg18875156 | -5.45 | 0.59 | 4.50E-10 | 10 |  |  |  |
| FGF5 | cg09956047 | -5.75 | 0.62 | 5.29E-10 | 16 | GNPTG | Body | Island |
| FGF5 | cg00007064 | -5.57 | 0.61 | 5.33E-10 | 9 | LHX6 | Body | N_Shore |
| FGF5 | cg01834185 | -5.97 | 0.65 | 5.33E-10 | 18 |  |  |  |
| FGF5 | cg12286158 | 3.79 | 0.41 | 6.46E-10 | 22 | DMC1 | TSS1500 | Island |
| FGF5 | cg07209071 | 5.96 | 0.65 | 6.59E-10 | 1 | PRDM16 | Body | N_Shelf |
| FGF5 | cg27173374 | -6.55 | 0.72 | 8.11E-10 | 14 | GNG2 | 5'UTR |  |
| FGF5 | cg01514538 | -6.52 | 0.72 | 1.28E-09 | 1 | FLJ42875 | Body | Island |
| FGF5 | cg09074856 | -8.61 | 0.96 | 1.32E-09 | 1 | FLJ42875 | Body | Island |
| FGF5 | cg10447827 | -15.58 | 1.74 | 1.41E-09 | 8 | DENND3 | Body |  |
| FGF5 | cg10767146 | 4.81 | 0.54 | 1.72E-09 | 15 | LRRK1 | Body | N_Shore |
| FGF5 | cg03278114 | -5.54 | 0.62 | 1.85E-09 | 5 | AGXT2L2 | TSS1500 | S_Shore |
| FGF5 | cg26993610 | 16.38 | 1.84 | 1.98E-09 | 1 | TSPAN2 | Body |  |
| FGF5 | cg01362990 | -5.88 | 0.66 | 2.00E-09 | 14 | FCF1 | TSS1500 | N_Shore |
| FGF5 | cg02915746 | 12.11 | 1.36 | 2.11E-09 | 2 | SLC30A3 | TSS1500 | Island |
| FGF5 | cg16387200 | -5.61 | 0.64 | 5.46E-09 | 7 |  |  |  |
| FGF5 | cg23250906 | -6.63 | 0.77 | 1.27E-08 | 1 | PPP1R12B | 3'UTR |  |
| FGF5 | cg06200777 | 14.18 | 1.66 | 1.28E-08 | 6 | SMPDL3A | 5'UTR | Island |
| FGF5 | cg27581373 | -2.59 | 0.3 | 1.34E-08 | 13 | INTS6 | 5'UTR |  |
| FGF5 | cg17299615 | 11.12 | 1.3 | 1.42E-08 | 19 |  |  | Island |
| FGF5 | cg17337840 | -2.58 | 0.3 | 1.68E-08 | 3 | OPA1 | Body; |  |
| FGF5 | cg27197651 | -5.4 | 0.65 | 3.87E-08 | 1 | FLJ42875 | Body | Island |
| FGF5 | cg02586023 | -7.26 | 0.87 | 4.12E-08 | 6 | FILIP1 | 5'UTR |  |
| FGF5 | cg01329098 | -5.84 | 0.7 | 5.54E-08 | 2 | RTN4 | Body; |  |
| FGF5 | cg14886676 | 11.17 | 1.35 | 5.91E-08 | 3 | C3orf21 | Body |  |
| FGF5 | cg07261749 | 23.51 | 2.86 | 1.02E-07 | 15 | LRRC49 | 5'UTR | S_Shore |
| FGF5 | cg09184192 | 13.13 | 1.66 | 4.28E-07 | 14 | OTUB2 | Body | Island |
| FGF5 | cg00995798 | 16.93 | 2.14 | 4.83E-07 | 2 | TTC27 | TSS1500 | N_Shore |
| FGF5 | cg26712947 | -5.66 | 0.72 | 7.27E-07 | 3 | LINC00885 | Body |  |
| FGF5 | cg02561823 | -5.79 | 0.76 | 2.62E-06 | 10 | CACNB2 | Body; |  |
| FGF5 | cg03695147 | 7.17 | 0.95 | 2.71E-06 | 16 | KIAA0895L | Body | Island |
| FGF5 | cg01587050 | 22.02 | 2.9 | 2.71E-06 | 17 | BRCA1 | TSS200 | N_Shore |
| FGF5 | cg03691418 | -4.85 | 0.64 | 3.12E-06 | 17 | B3GNTL1 | Body | Island |
| FGF5 | cg00116882 | 23.38 | 3.1 | 3.18E-06 | 12 | PIANP | TSS200; | S_Shore |
| FGF5 | cg04204526 | -10.01 | 1.33 | 3.35E-06 | 1 | FLJ42875 | Body; | S_Shore |
| FGF5 | cg04658354 | 25.95 | 3.48 | 4.97E-06 | 17 | NBR2 | TSS200 | N_Shore |
| FGF5 | cg23528723 | 4.42 | 0.59 | 5.66E-06 | 4 | LRPAP1 | TSS200 | Island |
| FGF5 | cg19620982 | 11.04 | 1.49 | 6.02E-06 | 12 | MSI1 | TSS1500 | S_Shore |
| FGF5 | cg15830081 | -5.14 | 0.69 | 6.19E-06 | 11 | TPH1 | Body |  |
| FGF5 | cg01546472 | -7.61 | 1.02 | 6.19E-06 | 4 | ODZ3 | 3'UTR | S_Shore |
| FGF5 | cg09831010 | 12.47 | 1.69 | 7.63E-06 | 17 | BRCA1 | TSS1500 | N_Shore |
| FGF5 | cg05354921 | 4.15 | 0.56 | 8.17E-06 | 1 | PRDM16 | Body; | N_Shelf |
| FGF5 | cg05125615 | -4.73 | 0.64 | 8.33E-06 | 6 | GMDS | Body; |  |
| FGF5 | cg14170557 | -4.5 | 0.61 | 8.92E-06 | 6 | SLC22A23 | Body; |  |
| FGF5 | cg02360445 | -11.74 | 1.62 | 1.39E-05 | 10 |  |  | Island |
| FGF5 | cg12182452 | 17.03 | 2.37 | 1.81E-05 | 17 | BRCA1 | TSS1500 | N_Shore |
| FGF5 | cg20601192 | -7.49 | 1.05 | 2.76E-05 | 2 | PDCD1 | Body | N_Shore |
| FGF5 | cg05082965 | 10.46 | 1.47 | 2.95E-05 | 14 |  |  | S_Shore |
| FGF5 | cg10852320 | 12.7 | 1.79 | 3.09E-05 | 5 |  |  | S_Shelf |
| FGF5 | cg18759102 | 5.25 | 0.75 | 5.42E-05 | 1 | PRDM16 | Body; | N_Shelf |
| FGF5 | cg10893007 | 31.42 | 4.48 | 5.50E-05 | 17 | NBR2 | TSS1500 | N_Shore |
| FGF5 | cg04944520 | -3.73 | 0.53 | 5.60E-05 | 8 | KHDRBS3 | Body |  |
| FGF5 | cg08846760 | -4.85 | 0.7 | 5.83E-05 | 16 | TRAPPC2L | TSS1500 | N_Shore |
| FGF5 | cg09110560 | -4.58 | 0.66 | 7.85E-05 | 21 | BACH1 | Body; |  |
| FGF5 | cg11529663 | 8.36 | 1.21 | 8.59E-05 | 17 | ST6GALNAC2 | TSS200 | Island |
| FGF5 | cg13595904 | -11.05 | 1.6 | 8.73E-05 | 3 | DZIP1L | TSS200 | Island |
| FGF5 | cg18065623 | -4.38 | 0.64 | 8.84E-05 | 4 | TMEM150C | Body |  |
| FGF5 | cg15995794 | 13.61 | 1.99 | 1.05E-04 | 6 | SMPDL3A | 5'UTR | Island |
| FGF5 | cg18428201 | -4.57 | 0.67 | 1.06E-04 | 8 | RGS20 | Body; |  |
| FGF5 | cg21262073 | 15.38 | 2.25 | 1.16E-04 | 19 | LSR | TSS200 | Island |
| FGF5 | cg19956540 | 2.17 | 0.32 | 1.16E-04 | 12 |  |  |  |
| FGF5 | cg15645888 | -4.43 | 0.65 | 1.36E-04 | 8 | FBXO16 | 3'UTR |  |
| FGF5 | cg21752583 | -2.54 | 0.38 | 1.57E-04 | 4 | LRPAP1 | Body | S_Shore |
| FGF5 | cg24336278 | 18.15 | 2.7 | 1.93E-04 | 1 | TP73 | TSS1500 | Island |
| FGF5 | cg17580798 | 4.86 | 0.73 | 2.25E-04 | 7 | MEST | TSS1500 | Island |
| FGF5 | cg27661381 | 20.74 | 3.1 | 2.27E-04 | 19 | PVRL2 | Body; | Island |
| FGF5 | cg05779048 | -3.33 | 0.5 | 2.50E-04 | 13 |  |  |  |
| FGF5 | cg16365799 | 13.62 | 2.05 | 2.96E-04 | 2 | DNER | 1stExon | Island |
| FGF5 | cg02325300 | 19.87 | 3 | 3.27E-04 | 12 | LMO3 | 5'UTR |  |
| FGF5 | cg21241317 | 12.61 | 1.91 | 3.68E-04 | 6 | PPT2 | Body; | S_Shore |
| FGF5 | cg14788420 | -2.32 | 0.36 | 5.94E-04 | 1 | MIR5096 | 5'UTR |  |
| FGF5 | cg04110421 | 13.59 | 2.08 | 5.94E-04 | 17 | NBR2 | TSS200 | N_Shore |
| FGF5 | cg05841627 | 14.56 | 2.25 | 6.41E-04 | 5 | TRIM7 | TSS1500 | Island |
| FGF5 | cg10112017 | 8.91 | 1.38 | 6.76E-04 | 4 |  |  | Island |
| FGF5 | cg12737520 | -2.71 | 0.42 | 7.30E-04 | 7 |  |  | N_Shelf |
| FGF5 | cg13050930 | 12.7 | 1.97 | 7.53E-04 | 6 | CDYL | TSS1500 | Island |
| FGF5 | cg23969554 | -11.67 | 1.82 | 8.87E-04 | 1 | FLJ42875 | Body | Island |
| FGF5 | cg17026879 | 19.02 | 2.98 | 9.54E-04 | 8 | NKX6-3 | Body | Island |
| FGF5 | cg14966901 | 4 | 0.63 | 9.94E-04 | 1 | PRDM16 | Body; | N_Shelf |
| FGF5 | cg15822346 | 11.79 | 1.85 | 1.10E-03 | 6 | SLC16A10 | TSS200 | Island |
| FGF5 | cg26911562 | 17.91 | 2.82 | 1.14E-03 | 5 | EIF4EBP3 | TSS200 | Island |
| FGF5 | cg02511231 | 6.81 | 1.09 | 1.75E-03 | 5 | TLX3 | Body | Island |
| FGF5 | cg23830653 | -13.16 | 2.1 | 1.83E-03 | 7 | PTPRN2 | Body; | S_Shore |
| FGF5 | cg19197515 | 18.16 | 2.92 | 2.23E-03 | 4 | PP12613 | TSS200 | Island |
| FGF5 | cg03199058 | 7.39 | 1.2 | 2.69E-03 | 1 | DNAH14 | 1stExon | Island |
| FGF5 | cg16630982 | 15.35 | 2.49 | 2.72E-03 | 17 | BRCA1 | 5'UTR | N_Shore |
| FGF5 | cg21306329 | 12.97 | 2.12 | 3.55E-03 | 17 | SLC16A5 | TSS200 | Island |
| FGF5 | cg18812980 | 18.12 | 2.96 | 3.58E-03 | 19 |  |  | Island |
| FGF5 | cg24022152 | -4.37 | 0.72 | 3.99E-03 | 11 | MICAL2 | 5'UTR | S_Shore |
| FGF5 | cg04159901 | 9.27 | 1.52 | 4.21E-03 | 1 |  |  | Island |
| FGF5 | cg18473652 | 25.59 | 4.23 | 4.89E-03 | 6 | COL12A1 | 5'UTR | N_Shore |
| FGF5 | cg19112780 | 27.18 | 4.49 | 5.81E-03 | 1 | PTPRU | 5'UTR | Island |
| FGF5 | cg01556502 | 13.44 | 2.24 | 5.88E-03 | 14 |  |  | Island |
| FGF5 | cg09551147 | 17.44 | 2.91 | 6.31E-03 | 10 | SORCS3 | TSS1500 | Island |
| FGF5 | cg01016660 | 11.95 | 1.99 | 6.37E-03 | 19 | KIAA1543 | 1stExon | Island |
| FGF5 | cg21747958 | 11.51 | 1.92 | 6.38E-03 | 19 | ZNF544 | 5'UTR | Island |
| FGF5 | cg19082920 | 24.08 | 4.01 | 6.38E-03 | 1 | LOC148709 | TSS200 | Island |
| FGF5 | cg26286408 | 13.89 | 2.33 | 6.98E-03 | 3 | CAND2 | Body; | Island |
| FGF5 | cg26667699 | 8.91 | 1.5 | 7.44E-03 | 5 | COL23A1 | TSS200 | Island |
| FGF5 | cg21155461 | 8.72 | 1.47 | 8.44E-03 | 19 | ZNF544 | 5'UTR | Island |
| FGF5 | cg05960081 | 17.7 | 3 | 9.08E-03 | 3 | ITGB5 | 5'UTR | Island |
| FGF5 | cg25284571 | 9.78 | 1.66 | 9.08E-03 | 15 |  |  |  |
| FGF5 | cg21077559 | 14.45 | 2.45 | 1.01E-02 | 4 | TMEM155 | 5'UTR | Island |
| FGF5 | cg18125479 | 13.93 | 2.37 | 1.07E-02 | 14 | PYGL | 1stExon | Island |
| FGF5 | cg00488323 | 17.02 | 2.9 | 1.13E-02 | 9 | DAB2IP | Body | Island |
| FGF5 | cg22417789 | 8.12 | 1.39 | 1.17E-02 | 13 | SPRY2 | TSS1500 | Island |
| FGF5 | cg24509300 | 6.92 | 1.18 | 1.32E-02 | 6 | PPT2 | Body; | S_Shore |
| FGF5 | cg07148752 | 17.16 | 2.95 | 1.36E-02 | 15 | TPM1 | TSS200 | Island |
| FGF5 | cg09384111 | 10.21 | 1.76 | 1.36E-02 | 9 | GLIS3 | 5'UTR | Island |
| FGF5 | cg07774879 | 30.03 | 5.15 | 1.36E-02 | 18 | PHLPP1 | 5'UTR | Island |
| FGF5 | cg10438011 | 13.67 | 2.35 | 1.39E-02 | 13 | SPRY2 | TSS1500 | Island |
| FGF5 | cg13384284 | -5.68 | 0.98 | 1.41E-02 | 3 |  |  | S_Shelf |
| FGF5 | cg20187250 | 15.93 | 2.72 | 1.42E-02 | 17 | BRCA1 | 5'UTR | N_Shore |
| FGF5 | cg05439349 | 2.51 | 0.43 | 1.43E-02 | 3 |  |  | S_Shelf |
| FGF5 | cg00412534 | 10.26 | 1.77 | 1.43E-02 | 2 |  |  | Island |
| FGF5 | cg14027333 | 4.36 | 0.75 | 1.44E-02 | 6 | PRRT1 | 3'UTR | N_Shore |
| FGF5 | cg04620091 | 5.91 | 1.02 | 1.46E-02 | 5 | NEUROG1 | TSS200 | Island |
| FGF5 | cg02522410 | 5.6 | 0.97 | 1.49E-02 | 10 | DOCK1 | TSS1500; | N_Shore |
| FGF5 | cg07814260 | 13.51 | 2.34 | 1.61E-02 | 8 | RGS20 | TSS200 | Island |
| FGF5 | cg01038484 | 4.46 | 0.77 | 1.61E-02 | 8 |  |  | Island |
| FGF5 | cg24718866 | 9.87 | 1.71 | 1.62E-02 | 2 | POMC | 5'UTR | Island |
| FGF5 | cg21253966 | 11.13 | 1.94 | 1.72E-02 | 17 | NBR2 | TSS200 | N_Shore |
| FGF5 | cg24053165 | 3.4 | 0.59 | 1.75E-02 | 10 |  |  | N_Shore |
| FGF5 | cg13667739 | 9.05 | 1.58 | 1.93E-02 | 14 | CRIP2 | Body | Island |
| FGF5 | cg10068989 | 9.97 | 1.74 | 1.99E-02 | 11 |  |  | Island |
| FGF5 | cg25583136 | -1.88 | 0.33 | 2.07E-02 | 11 | MS4A6E | Body |  |
| FGF5 | cg08397064 | 18.47 | 3.23 | 2.08E-02 | 1 | FNBP1L | TSS200 | N_Shore |
| FGF5 | cg27070869 | 6.41 | 1.12 | 2.16E-02 | 6 | PPT2 | Body; | S_Shore |
| FGF5 | cg03009363 | 12.54 | 2.21 | 2.16E-02 | 6 | TFAP2A | Body | Island |
| FGF5 | cg05648672 | 11.09 | 1.95 | 2.29E-02 | 5 | MEGF10 | TSS1500 | N_Shore |
| FGF5 | cg26165269 | 15.45 | 2.72 | 2.51E-02 | 17 |  |  | Island |
| FGF5 | cg08640609 | 5.62 | 1 | 2.76E-02 | 1 | CAMTA1 | Body | Island |
| FGF5 | cg00713411 | 9.92 | 1.76 | 2.76E-02 | 8 | PNMA2 | TSS1500 | Island |
| FGF5 | cg12473285 | 5.79 | 1.03 | 2.80E-02 | 17 | SOST | Body | Island |
| FGF5 | cg11325420 | -3.81 | 0.68 | 2.85E-02 | 18 | RPRD1A | TSS1500 | S_Shore |
| FGF5 | cg15079268 | 18.62 | 3.32 | 2.98E-02 | 8 | ASPH | TSS200 | Island |
| FGF5 | cg13507964 | 13.02 | 2.32 | 2.98E-02 | 21 | MX1 | 5'UTR | Island |
| FGF5 | cg02532538 | 5.58 | 0.99 | 2.98E-02 | 17 |  |  | Island |
| FGF5 | cg03307401 | 11.08 | 1.98 | 3.02E-02 | 19 | KLK13 | Body | Island |
| FGF5 | cg18975376 | 12.26 | 2.19 | 3.04E-02 | 10 | BAMBI | Body | Island |
| FGF5 | cg09555914 | 26.1 | 4.66 | 3.10E-02 | 19 | ZNF773 | 5'UTR | Island |
| FGF5 | cg25052312 | 10.81 | 1.93 | 3.21E-02 | 15 |  |  | N_Shore |
| FGF5 | cg18649745 | 25.52 | 4.54 | 3.35E-02 | 19 | ZNF350 | TSS200 |  |
| FGF5 | cg03768318 | 19.04 | 3.4 | 3.44E-02 | 16 | CCDC113 | Body; | S_Shore |
| FGF5 | cg15080430 | 5.99 | 1.07 | 3.45E-02 | 6 | COL19A1 | Body |  |
| FGF5 | cg10600783 | 9.98 | 1.79 | 3.45E-02 | 6 | ZNF318 | Body | Island |
| FGF5 | cg06741060 | -2.82 | 0.51 | 3.45E-02 | 7 |  |  |  |
| FGF5 | cg19531713 | 10.28 | 1.84 | 3.49E-02 | 17 | BRCA1 | 5'UTR | N_Shore |
| FGF5 | cg22967080 | -4.79 | 0.86 | 3.55E-02 | 14 | GNG2 | 5'UTR |  |
| FGF5 | cg16963062 | 13.47 | 2.42 | 3.55E-02 | 17 | BRCA1 | 5'UTR | N_Shore |
| FGF5 | cg08245274 | 6.89 | 1.24 | 3.60E-02 | 7 | DGKI | 1stExon | Island |
| FGF5 | cg10003549 | 14.94 | 2.69 | 3.70E-02 | 20 | KIF16B | TSS200 | Island |
| FGF5 | cg13874739 | 11.88 | 2.14 | 3.76E-02 | 11 |  |  | Island |
| FGF5 | cg26325444 | 6.88 | 1.24 | 3.94E-02 | 3 | CCDC50 | 5'UTR | Island |
| FGF5 | cg20737185 | 6.02 | 1.09 | 4.09E-02 | 6 | C6orf186 | TSS200 | Island |
| FGF5 | cg09252999 | 17.17 | 3.11 | 4.29E-02 | 11 | PAX6 | 5'UTR | Island |
| FGF5 | cg11990980 | 4.01 | 0.73 | 4.41E-02 | 15 | PCSK6 | 1stExon | Island |
| FGF5 | cg19801421 | 9.56 | 1.74 | 4.46E-02 | 21 | NRIP1 | 5'UTR | Island |
| FGF5 | cg20121142 | 12.02 | 2.19 | 4.46E-02 | 7 | TWIST1 | TSS1500 | Island |
| FGF5 | cg01644850 | 26.41 | 4.8 | 4.46E-02 | 19 | ZNF551 | TSS200 | N_Shore |
| FGF5 | cg26438929 | 18.05 | 3.28 | 4.46E-02 | 15 |  |  |  |
| FGF5 | cg22587479 | 3.3 | 0.6 | 4.46E-02 | 2 | WNT6 | Body | Island |
| FGF5 | cg22083798 | 5.86 | 1.07 | 4.46E-02 | 7 | IGFBP3 | TSS1500; | S_Shore |
| FGF5 | cg21656751 | 5.79 | 1.05 | 4.48E-02 | 7 | HECW1 | 5'UTR | Island |
| FGF5 | cg26185347 | 17.24 | 3.14 | 4.48E-02 | 19 | LSR | TSS200 | Island |
| FGF5 | cg05045130 | 11.07 | 2.02 | 4.50E-02 | 10 |  |  | Island |
| FGF5 | cg00287312 | 6.71 | 1.23 | 4.84E-02 | 13 | PCDH8 | 1stExon | Island |
| FGF5 | cg14935271 | 7.42 | 1.36 | 4.99E-02 | 12 | PAWR | Body | Island |
| FGF23 | cg02095624 | -6 | 0.64 | 1.77E-09 | 10 | AKR1C8P | Body |  |
| FGF23 | cg19153563 | -3.14 | 0.34 | 1.77E-09 | 15 | LINC01169 | Body |  |
| FGF23 | cg11670074 | -5.43 | 0.59 | 1.77E-09 | 15 |  |  | S_Shelf |
| FGF23 | cg00004255 | -6.33 | 0.69 | 1.77E-09 | 21 | LOC101927843 | Body |  |
| FGF23 | cg22000834 | -6.19 | 0.67 | 1.77E-09 | 19 | ZNF573 | 5'UTR |  |
| FGF23 | cg05140425 | -5.53 | 0.6 | 1.77E-09 | 1 |  |  |  |
| FGF23 | cg06257454 | -2.93 | 0.32 | 1.77E-09 | 13 |  |  | N_Shelf |
| FGF23 | cg12178225 | -2.87 | 0.32 | 2.12E-09 | 6 | TRIM26 | Body |  |
| FGF23 | cg17985746 | -5.66 | 0.63 | 2.53E-09 | 6 | C6orf170 | Body |  |
| FGF23 | cg21383646 | -5.97 | 0.66 | 2.74E-09 | 8 | TACC1 | 5'UTR |  |
| FGF23 | cg26459904 | -5.27 | 0.59 | 3.51E-09 | 1 | ROR1 | Body; |  |
| FGF23 | cg08258915 | -5.61 | 0.63 | 4.02E-09 | 6 |  |  |  |
| FGF23 | cg02584802 | -5.6 | 0.63 | 4.02E-09 | 11 |  |  | S_Shelf |
| FGF23 | cg03577153 | 5.59 | 0.63 | 6.81E-09 | 19 | ZNF350 | TSS200 |  |
| FGF23 | cg26360953 | -5.96 | 0.68 | 7.34E-09 | 1 |  |  |  |
| FGF23 | cg24654233 | -5.14 | 0.59 | 8.70E-09 | 7 | RADIL | Body | S_Shelf |
| FGF23 | cg21886316 | 26.01 | 2.99 | 1.10E-08 | 8 | MED30 | 5'UTR | Island |
| FGF23 | cg19052142 | -5.42 | 0.62 | 1.17E-08 | 10 | ANXA11 | ExonBnd |  |
| FGF23 | cg03438346 | -5.81 | 0.67 | 1.36E-08 | 18 | TTC39C | TSS1500 |  |
| FGF23 | cg27461278 | -5.64 | 0.66 | 1.75E-08 | 6 |  |  |  |
| FGF23 | cg26715704 | -4.51 | 0.53 | 2.13E-08 | 20 |  |  |  |
| FGF23 | cg23387597 | -4.91 | 0.58 | 3.02E-08 | 10 | ITPRIP | TSS200 | N_Shelf |
| FGF23 | cg09616608 | -4.83 | 0.57 | 3.23E-08 | 16 | LMF1 | Body | N_Shore |
| FGF23 | cg17006098 | -5.37 | 0.64 | 3.64E-08 | 17 | RAB11FIP4 | Body; |  |
| FGF23 | cg17317548 | -5.91 | 0.7 | 3.64E-08 | 5 | UBE2B | 3'UTR |  |
| FGF23 | cg20954746 | -6.41 | 0.76 | 3.83E-08 | 2 | DGKD | TSS1500 |  |
| FGF23 | cg05160935 | 31.45 | 3.76 | 5.55E-08 | 8 | MED30 | TSS1500 | N_Shore |
| FGF23 | cg12322015 | 3.1 | 0.37 | 5.55E-08 | 6 | ECI2 | TSS1500 | Island |
| FGF23 | cg12914817 | -12.11 | 1.52 | 5.60E-07 | 5 | KCNIP1 | 5'UTR |  |
| FGF23 | cg06170341 | -5.18 | 0.66 | 8.55E-07 | 10 | FGFR2 | Body |  |
| FGF23 | cg12421899 | -4.56 | 0.58 | 1.02E-06 | 19 |  |  | S_Shelf |
| FGF23 | cg17473624 | -5.81 | 0.74 | 1.11E-06 | 10 |  |  |  |
| FGF23 | cg14010086 | -13.39 | 1.72 | 1.11E-06 | 17 | B4GALNT2 | Body |  |
| FGF23 | cg21566278 | -4.79 | 0.62 | 1.43E-06 | 13 | C13orf31 | 3'UTR |  |
| FGF23 | cg15954386 | 8.48 | 1.11 | 2.40E-06 | 4 | DDX60 | TSS200 | S_Shore |
| FGF23 | cg18832251 | 10.41 | 1.37 | 3.08E-06 | 4 | DDX60 | TSS200 | S_Shore |
| FGF23 | cg17464298 | -3.36 | 0.44 | 3.20E-06 | 16 |  |  |  |
| FGF23 | cg26883238 | -3.95 | 0.52 | 4.01E-06 | 10 | FAM149B1 | Body |  |
| FGF23 | cg05306314 | 15.73 | 2.09 | 4.59E-06 | 12 | PTPRR | 5'UTR |  |
| FGF23 | cg06050213 | -4.94 | 0.66 | 4.63E-06 | 3 | KCNAB1 | Body |  |
| FGF23 | cg17878237 | -11.96 | 1.6 | 6.11E-06 | 2 |  |  |  |
| FGF23 | cg19252175 | 8.12 | 1.09 | 6.51E-06 | 4 | DDX60 | TSS200 | S_Shore |
| FGF23 | cg03099590 | -5.52 | 0.74 | 7.98E-06 | 10 | LOXL4 | Body |  |
| FGF23 | cg15091264 | -14.33 | 1.94 | 9.33E-06 | 16 | CDH13 | Body |  |
| FGF23 | cg08099701 | -9.08 | 1.23 | 9.86E-06 | 15 | GABRA5 | Body |  |
| FGF23 | cg18695143 | -2.89 | 0.4 | 1.50E-05 | 16 | CBFA2T3 | 5'UTR | N_Shelf |
| FGF23 | cg01824305 | 8.14 | 1.12 | 1.64E-05 | 4 | DDX60 | TSS200 | S_Shore |
| FGF23 | cg03835941 | -4.99 | 0.69 | 1.99E-05 | 16 |  |  |  |
| FGF23 | cg05883164 | -6.05 | 0.84 | 2.76E-05 | 2 |  |  |  |
| FGF23 | cg19196085 | -9.66 | 1.35 | 2.78E-05 | 21 |  |  |  |
| FGF23 | cg26704506 | 9.83 | 1.38 | 3.36E-05 | 4 | DDX60 | TSS200 | S_Shore |
| FGF23 | cg05422925 | -2.33 | 0.33 | 3.42E-05 | 12 |  |  |  |
| FGF23 | cg20741319 | -5.01 | 0.71 | 4.21E-05 | 3 | WNT5A | Body | N_Shelf |
| FGF23 | cg14232889 | -13.74 | 1.95 | 4.87E-05 | 12 | TEAD4 | 5'UTR |  |
| FGF23 | cg19476037 | -9.74 | 1.39 | 5.92E-05 | 6 | SMOC2 | Body | N_Shore |
| FGF23 | cg04454603 | -10.19 | 1.45 | 5.92E-05 | 2 |  |  |  |
| FGF23 | cg17346145 | 5.56 | 0.8 | 6.13E-05 | 17 | CBX2 | Body | N_Shelf |
| FGF23 | cg05641033 | 25.04 | 3.57 | 6.13E-05 | 12 |  |  | Island |
| FGF23 | cg05250615 | -11.39 | 1.63 | 6.52E-05 | 1 |  |  |  |
| FGF23 | cg24579320 | -8.99 | 1.3 | 7.83E-05 | 16 | HYDIN | Body |  |
| FGF23 | cg07736135 | -3.84 | 0.56 | 9.36E-05 | 5 | ATP6AP1L | Body |  |
| FGF23 | cg11041239 | -2.13 | 0.31 | 9.95E-05 | 8 |  |  | S_Shelf |
| FGF23 | cg07754218 | -4.3 | 0.63 | 1.10E-04 | 2 | ABI2 | TSS200 |  |
| FGF23 | cg03968041 | -7.15 | 1.04 | 1.10E-04 | 10 | JAKMIP3 | 3'UTR |  |
| FGF23 | cg10221266 | -10.02 | 1.46 | 1.12E-04 | 7 |  |  | N_Shelf |
| FGF23 | cg16144367 | 25.23 | 3.7 | 1.42E-04 | 1 | HEATR1 | TSS1500 | S_Shore |
| FGF23 | cg22288012 | -8.79 | 1.29 | 1.42E-04 | 8 |  |  |  |
| FGF23 | cg13386109 | -4.7 | 0.69 | 1.44E-04 | 6 |  |  | S_Shore |
| FGF23 | cg26807538 | -4.59 | 0.68 | 1.44E-04 | 12 |  |  |  |
| FGF23 | cg05408896 | -5.09 | 0.75 | 1.47E-04 | 3 |  |  |  |
| FGF23 | cg11697412 | -4.52 | 0.67 | 1.59E-04 | 6 |  |  | S_Shelf |
| FGF23 | cg06808825 | -11.35 | 1.68 | 1.72E-04 | 4 |  |  |  |
| FGF23 | cg07215869 | -3.54 | 0.53 | 2.15E-04 | 10 | ADARB2 | Body |  |
| FGF23 | cg07305850 | -18.04 | 2.69 | 2.19E-04 | 3 |  |  | Island |
| FGF23 | cg12888141 | -9.49 | 1.41 | 2.20E-04 | 10 |  |  |  |
| FGF23 | cg22060073 | 6 | 0.9 | 2.43E-04 | 8 | ADCY8 | TSS200 | Island |
| FGF23 | cg21340660 | -7.92 | 1.19 | 2.75E-04 | 16 |  |  |  |
| FGF23 | cg00490349 | -10.02 | 1.51 | 3.03E-04 | 11 |  |  |  |
| FGF23 | cg17737875 | -12.4 | 1.87 | 3.32E-04 | 7 | PTPRN2 | Body | N_Shore |
| FGF23 | cg06163425 | 6.91 | 1.04 | 3.43E-04 | 10 | EBF3 | Body | N_Shore |
| FGF23 | cg26538349 | 6.73 | 1.02 | 3.55E-04 | 4 | DDX60 | 5'UTR | S_Shore |
| FGF23 | cg02422627 | 12.62 | 1.93 | 4.84E-04 | 8 | MED30 | 5'UTR | Island |
| FGF23 | cg07196462 | -12.37 | 1.9 | 5.55E-04 | 2 |  |  |  |
| FGF23 | cg02189001 | -7.4 | 1.14 | 5.60E-04 | 14 | OR5AU1 | 1stExon |  |
| FGF23 | cg15209885 | 6.87 | 1.05 | 5.73E-04 | 17 | CBX2 | Body; | S_Shore |
| FGF23 | cg02661221 | -10.54 | 1.62 | 6.01E-04 | 14 |  |  |  |
| FGF23 | cg13882311 | -7.72 | 1.19 | 6.94E-04 | 9 | OLFM1 | Body; | S_Shelf |
| FGF23 | cg08375775 | 3.83 | 0.59 | 6.94E-04 | 4 | DDX60 | 5'UTR | Island |
| FGF23 | cg14171333 | -2.96 | 0.46 | 7.27E-04 | 11 | ART1 | 5'UTR |  |
| FGF23 | cg01345415 | -27 | 4.19 | 7.30E-04 | 14 | OR4E2 | 1stExon |  |
| FGF23 | cg02287146 | -9.34 | 1.45 | 7.41E-04 | 5 |  |  |  |
| FGF23 | cg20858149 | 6.38 | 0.99 | 7.69E-04 | 1 | NKAIN1 | TSS200 | S_Shore |
| FGF23 | cg06111534 | -6.96 | 1.08 | 7.69E-04 | 19 |  |  |  |
| FGF23 | cg04074010 | -3.29 | 0.51 | 7.97E-04 | 10 |  |  |  |
| FGF23 | cg21837864 | -3.51 | 0.55 | 8.51E-04 | 10 | NPFFR1 | Body |  |
| FGF23 | cg27048363 | -6.73 | 1.05 | 9.04E-04 | 5 |  |  |  |
| FGF23 | cg02625318 | -2.9 | 0.45 | 9.48E-04 | 3 | SLC6A6 | Body; |  |
| FGF23 | cg07892579 | -2.62 | 0.41 | 1.03E-03 | 8 |  |  |  |
| FGF23 | cg20898358 | 2.77 | 0.44 | 1.18E-03 | 17 | MPRIP | Body; | S_Shore |
| FGF23 | cg27342910 | -5.8 | 0.92 | 1.40E-03 | 4 |  |  |  |
| FGF23 | cg16329944 | -8.95 | 1.42 | 1.40E-03 | 14 | SLC25A21 | Body; |  |
| FGF23 | cg19079570 | 4.04 | 0.64 | 1.56E-03 | 4 | DDX60 | 5'UTR | Island |
| FGF23 | cg20141018 | -7.25 | 1.16 | 1.57E-03 | 4 |  |  |  |
| FGF23 | cg14595626 | -11.54 | 1.84 | 1.72E-03 | 10 | CCNYL2 | TSS1500 | N_Shelf |
| FGF23 | cg09520498 | -7.06 | 1.13 | 1.84E-03 | 7 | EIF3IP1 | TSS1500 |  |
| FGF23 | cg14958265 | -10.88 | 1.75 | 2.28E-03 | 12 | NUAK1 | Body |  |
| FGF23 | cg06859969 | 9.42 | 1.52 | 2.34E-03 | 4 |  |  | S_Shore |
| FGF23 | cg10035564 | 12.01 | 1.94 | 2.37E-03 | 12 |  |  | S_Shore |
| FGF23 | cg15259931 | -13.41 | 2.17 | 2.63E-03 | 1 |  |  |  |
| FGF23 | cg21327483 | -6.82 | 1.11 | 2.63E-03 | 8 |  |  |  |
| FGF23 | cg16858222 | -5.4 | 0.88 | 2.73E-03 | 8 |  |  |  |
| FGF23 | cg20742784 | 5.99 | 0.97 | 2.73E-03 | 5 | CCL28 | TSS200 | S_Shore |
| FGF23 | cg17687247 | 14.1 | 2.29 | 2.73E-03 | 4 |  |  | N_Shore |
| FGF23 | cg14044930 | -3.47 | 0.56 | 2.83E-03 | 14 |  |  |  |
| FGF23 | cg11138203 | -7.93 | 1.29 | 3.09E-03 | 8 |  |  |  |
| FGF23 | cg17198047 | -2.85 | 0.46 | 3.18E-03 | 1 | PLD5 | Body; |  |
| FGF23 | cg03836052 | -6.49 | 1.06 | 3.36E-03 | 4 |  |  |  |
| FGF23 | cg11364888 | -6.98 | 1.15 | 3.48E-03 | 12 |  |  |  |
| FGF23 | cg08222293 | -2.78 | 0.46 | 3.78E-03 | 5 | TRIM7 | 5'UTR | N_Shelf |
| FGF23 | cg15464689 | -4.16 | 0.68 | 3.78E-03 | 14 |  |  |  |
| FGF23 | cg14421700 | 5.28 | 0.87 | 4.39E-03 | 17 | CBX2 | 3'UTR | N_Shelf |
| FGF23 | cg07612728 | 6.83 | 1.13 | 4.90E-03 | 20 | LINC00261 | Body | N_Shore |
| FGF23 | cg24821952 | -11 | 1.83 | 5.19E-03 | 17 |  |  |  |
| FGF23 | cg10640435 | 8.77 | 1.46 | 5.51E-03 | 1 | HAPLN2 | Body | Island |
| FGF23 | cg21467904 | 38.62 | 6.46 | 7.08E-03 | 8 | MED30 | TSS200 | N_Shore |
| FGF23 | cg23002384 | 15.34 | 2.58 | 7.25E-03 | 1 |  |  | S_Shore |
| FGF23 | cg03734156 | -6 | 1.01 | 7.78E-03 | 2 |  |  |  |
| FGF23 | cg27024922 | 9.83 | 1.66 | 7.78E-03 | 9 | POMT1 | TSS1500 | N_Shore |
| FGF23 | cg27295571 | -6.42 | 1.08 | 7.82E-03 | 20 | DEFB122 | TSS1500 |  |
| FGF23 | cg22724765 | 5.93 | 1 | 8.31E-03 | 5 | CCL28 | Body | S_Shore |
| FGF23 | cg14470187 | -12.92 | 2.2 | 9.19E-03 | 19 |  |  |  |
| FGF23 | cg23664833 | -6.04 | 1.03 | 9.40E-03 | 4 |  |  |  |
| FGF23 | cg05946669 | -2.25 | 0.38 | 9.40E-03 | 11 |  |  |  |
| FGF23 | cg09898554 | -4.19 | 0.72 | 9.89E-03 | 18 | NETO1 | Body; |  |
| FGF23 | cg22057372 | -10.57 | 1.8 | 9.89E-03 | 7 | PTPRN2 | Body; |  |
| FGF23 | cg03791005 | 6.33 | 1.08 | 1.04E-02 | 16 |  |  | Island |
| FGF23 | cg15601946 | 9.4 | 1.61 | 1.10E-02 | 21 | KCNJ6 | 5'UTR | N_Shore |
| FGF23 | cg00918738 | -2.88 | 0.5 | 1.19E-02 | 20 | TCF15 | Body | N_Shore |
| FGF23 | cg15367245 | 5.62 | 0.97 | 1.43E-02 | 1 | NKAIN1 | TSS200 | S_Shore |
| FGF23 | cg25221196 | -4.61 | 0.8 | 1.50E-02 | 1 |  |  | Island |
| FGF23 | cg12591898 | -10.1 | 1.75 | 1.50E-02 | 7 | RNF32 | TSS1500 | S_Shelf |
| FGF23 | cg23871081 | -13.84 | 2.41 | 1.67E-02 | 10 |  |  | N_Shore |
| FGF23 | cg08708644 | 6.01 | 1.05 | 1.68E-02 | 5 | CCL28 | TSS200 | S_Shore |
| FGF23 | cg04193143 | -7.21 | 1.26 | 1.73E-02 | 6 |  |  |  |
| FGF23 | cg20403795 | -5.57 | 0.97 | 1.73E-02 | 4 | LOC101928978 | Body |  |
| FGF23 | cg20398123 | -10.65 | 1.85 | 1.76E-02 | 21 | C21orf29 | Body | S_Shore |
| FGF23 | cg25114930 | -5.43 | 0.95 | 1.76E-02 | 4 |  |  |  |
| FGF23 | cg07015485 | -4.75 | 0.84 | 2.15E-02 | 10 | CUZD1 | Body |  |
| FGF23 | cg17850597 | -10.24 | 1.8 | 2.19E-02 | 19 | OR10H1 | 1stExon | N_Shore |
| FGF23 | cg03867963 | -8.82 | 1.55 | 2.19E-02 | 2 | CCDC85A | Body |  |
| FGF23 | cg03974415 | -11.67 | 2.06 | 2.24E-02 | 5 |  |  |  |
| FGF23 | cg22019305 | -10.55 | 1.86 | 2.26E-02 | 6 | TTLL2 | Body |  |
| FGF23 | cg18093572 | -8.29 | 1.47 | 2.39E-02 | 5 |  |  |  |
| FGF23 | cg02805410 | -5.33 | 0.94 | 2.45E-02 | 1 | MIR1273E | Body |  |
| FGF23 | cg04436077 | -5.59 | 0.99 | 2.79E-02 | 3 | ATP2B2 | 5'UTR |  |
| FGF23 | cg21623854 | -2.82 | 0.5 | 2.84E-02 | 3 |  |  |  |
| FGF23 | cg05572751 | -7.15 | 1.27 | 2.88E-02 | 8 |  |  |  |
| FGF23 | cg19828457 | -3.44 | 0.61 | 3.14E-02 | 8 |  |  |  |
| FGF23 | cg26281844 | -3.62 | 0.65 | 3.18E-02 | 8 | MROH5 | Body; |  |
| FGF23 | cg25753047 | -4.65 | 0.83 | 3.23E-02 | 11 |  |  |  |
| FGF23 | cg04026492 | 10.62 | 1.9 | 3.25E-02 | 9 |  |  | Island |
| FGF23 | cg09999998 | -7.84 | 1.41 | 3.28E-02 | 22 | SEZ6L | Body; |  |
| FGF23 | cg02662180 | -3.96 | 0.71 | 3.38E-02 | 12 | BRI3BP | Body | S_Shelf |
| FGF23 | cg05611414 | -2.36 | 0.42 | 3.43E-02 | 19 | SNAR-D | TSS200 |  |
| FGF23 | cg00089486 | 5.1 | 0.92 | 3.68E-02 | 3 | SHOX2 | Body; | S_Shore |
| FGF23 | cg15119959 | -6.73 | 1.22 | 3.99E-02 | 3 |  |  |  |
| FGF23 | cg09074450 | -5.41 | 0.98 | 4.01E-02 | 16 |  |  | N_Shore |
| FGF23 | cg07874330 | -2.8 | 0.51 | 4.01E-02 | 1 |  |  |  |
| FGF23 | cg25443566 | -2.87 | 0.52 | 4.03E-02 | 15 |  |  |  |
| FGF23 | cg11784799 | -7.44 | 1.35 | 4.04E-02 | 8 |  |  |  |
| FGF23 | cg13198594 | 4.18 | 0.76 | 4.08E-02 | 4 | DDX60 | 5'UTR | Island |
| FGF23 | cg12275827 | -6.95 | 1.26 | 4.11E-02 | 10 | BTBD16 | Body |  |
| FGF23 | cg05566388 | -4.85 | 0.88 | 4.11E-02 | 9 | GDA | 5'UTR |  |
| FGF23 | cg10774701 | -10.27 | 1.87 | 4.71E-02 | 2 | PDE11A | TSS200 | N_Shelf |
| FGF23 | cg19521564 | 4.88 | 0.89 | 4.73E-02 | 4 | DDX60 | 5'UTR | Island |
| FGF23 | cg05392435 | -5.07 | 0.93 | 4.91E-02 | 6 |  |  | S_Shelf |
| IL6 | cg26049527 | -5.61 | 0.78 | 1.43E-03 | 1 | LHX4 | Body |  |
| IL6 | cg07697887 | -12.77 | 1.84 | 1.76E-03 | 11 |  |  | S_Shore |
| IL6 | cg09826539 | -6.57 | 0.96 | 1.76E-03 | 8 | TTI2 | Body; | N_Shelf |
| IL6 | cg16362378 | -10.96 | 1.6 | 1.76E-03 | 6 | VPS52 | Body | S_Shore |
| IL6 | cg07213202 | 17.33 | 2.56 | 2.26E-03 | 7 | ELFN1 | 5'UTR | N_Shore |
| IL6 | cg10477088 | 11.24 | 1.68 | 2.60E-03 | 22 |  |  | Island |
| IL6 | cg15731435 | -5.72 | 0.86 | 2.60E-03 | 19 | KCNJ14 | 5'UTR | N_Shelf |
| IL6 | cg06932125 | -10.5 | 1.58 | 2.60E-03 | 12 | COQ10A | TSS1500; | N_Shore |
| IL6 | cg25622125 | -11.95 | 1.81 | 3.30E-03 | 15 | HERC2 | Body | S_Shelf |
| IL6 | cg02074074 | -5.23 | 0.8 | 4.47E-03 | 12 | GPR133 | 3'UTR | S_Shore |
| IL6 | cg16216934 | -9.48 | 1.46 | 4.68E-03 | 2 | USP34 | Body |  |
| IL6 | cg13632959 | 10.15 | 1.57 | 4.68E-03 | 10 | FAS | TSS1500 | S_Shore |
| IL6 | cg16563470 | -5.61 | 0.87 | 4.68E-03 | 17 | CNP | Body | Island |
| IL6 | cg12944704 | -5.22 | 0.81 | 4.68E-03 | 3 | ADAMTS9-AS1 | TSS1500 |  |
| IL6 | cg25372278 | 10.91 | 1.7 | 5.16E-03 | 11 | CALCB | 5'UTR | S_Shore |
| IL6 | cg01963059 | -11.01 | 1.72 | 5.16E-03 | 1 | C1orf161 | Body |  |
| IL6 | cg24415717 | -12.21 | 1.91 | 5.16E-03 | 7 | RBM33 | Body |  |
| IL6 | cg06726797 | -11.18 | 1.77 | 7.57E-03 | 1 | LRRC42 | Body; |  |
| IL6 | cg17329287 | 5.75 | 0.91 | 7.83E-03 | 1 | RNF220 | TSS1500 | N_Shore |
| IL6 | cg26988628 | -11.98 | 1.91 | 7.97E-03 | 3 | TNIK | Body; |  |
| IL6 | cg02981663 | -9.96 | 1.6 | 1.06E-02 | 13 | POLR1D | Body |  |
| IL6 | cg13435651 | 9.41 | 1.53 | 1.27E-02 | 22 |  |  | Island |
| IL6 | cg02810627 | -9.64 | 1.58 | 1.68E-02 | 11 |  |  |  |
| IL6 | cg02992617 | -10.54 | 1.75 | 2.29E-02 | 9 | NR5A1 | Body | N_Shore |
| IL6 | cg19133023 | -9.28 | 1.54 | 2.29E-02 | 11 | CHID1 | Body; | S_Shore |
| IL6 | cg19343518 | -9.44 | 1.57 | 2.29E-02 | 6 | ARID1B | Body; |  |
| IL6 | cg08365067 | -10.76 | 1.79 | 2.45E-02 | 17 | GAS7 | Body; |  |
| IL6 | cg15418275 | -8.63 | 1.45 | 2.71E-02 | 9 |  |  |  |
| IL6 | cg03997678 | -12.47 | 2.09 | 2.71E-02 | 1 | LINC01226 | Body |  |
| IL6 | cg15108047 | -8.73 | 1.46 | 2.71E-02 | 20 |  |  | S_Shelf |
| IL6 | cg10163262 | -8.83 | 1.48 | 2.71E-02 | 1 |  |  | S_Shelf |
| IL6 | cg12836499 | -10.23 | 1.73 | 3.26E-02 | 2 | LOC105616981 | 5'UTR |  |
| IL6 | cg13026437 | -9.49 | 1.62 | 3.75E-02 | 15 |  |  |  |
| IL6 | cg13372624 | -9.04 | 1.55 | 4.65E-02 | 13 | TEX29 | Body; |  |
| IL10RA | cg08685054 | -11.42 | 1.26 | 2.00E-08 | 8 |  |  |  |
| IL10RA | cg08821726 | -9.62 | 1.12 | 1.03E-07 | 12 | ERC1 | Body; |  |
| IL10RA | cg03062648 | -4.95 | 0.58 | 1.03E-07 | 6 | PPP1R11 | Body | N_Shelf |
| IL10RA | cg26444951 | 9.62 | 1.13 | 1.03E-07 | 4 |  |  | N_Shore |
| IL10RA | cg08605554 | -4.96 | 0.58 | 1.03E-07 | 19 | VRK3 | Body; |  |
| IL10RA | cg08112248 | -4.89 | 0.58 | 1.03E-07 | 6 | SMOC2 | Body; |  |
| IL10RA | cg05147262 | -11.03 | 1.3 | 1.03E-07 | 4 | ENAM | Body |  |
| IL10RA | cg20929551 | -4.83 | 0.57 | 1.03E-07 | 15 | C15orf33 | Body |  |
| IL10RA | cg23652363 | -9.4 | 1.12 | 1.03E-07 | 11 |  |  |  |
| IL10RA | cg20964929 | -9.25 | 1.1 | 1.22E-07 | 19 | MAU2 | Body |  |
| IL10RA | cg20772292 | -9.62 | 1.15 | 1.27E-07 | 1 | RASSF5 | Body; |  |
| IL10RA | cg02053920 | -5.02 | 0.6 | 1.30E-07 | 6 | PSORS1C1 | 5'UTR |  |
| IL10RA | cg22832660 | -10.07 | 1.21 | 1.30E-07 | 7 | ST7 | Body; |  |
| IL10RA | cg11525242 | -6.1 | 0.73 | 1.30E-07 | 16 | GTF3C1 | Body; |  |
| IL10RA | cg19104976 | -10 | 1.22 | 2.11E-07 | 5 | PHF15 | Body |  |
| IL10RA | cg14050200 | -8.88 | 1.09 | 2.64E-07 | 1 | SMYD3 | Body; |  |
| IL10RA | cg10769343 | 5.85 | 0.72 | 3.88E-07 | 14 | PSME2 | TSS1500 | S_Shore |
| IL10RA | cg08353119 | -10.17 | 1.26 | 4.17E-07 | 1 |  |  |  |
| IL10RA | cg20032501 | -4.6 | 0.57 | 4.56E-07 | 11 | CCDC89 | 3'UTR |  |
| IL10RA | cg10526630 | -8.9 | 1.11 | 4.60E-07 | 15 | DAPK2 | Body |  |
| IL10RA | cg01600402 | -9.88 | 1.23 | 4.60E-07 | 22 | CRYBB1 | Body |  |
| IL10RA | cg11743470 | -10.04 | 1.27 | 9.54E-07 | 5 | GCNT4 | TSS1500 | N_Shelf |
| IL10RA | cg19287064 | -8.26 | 1.05 | 1.03E-06 | 17 | RPTOR | Body; |  |
| IL10RA | cg12161132 | -14.4 | 1.86 | 2.21E-06 | 10 | STK32C | Body |  |
| IL10RA | cg09898650 | -9.62 | 1.24 | 2.21E-06 | 5 | CNOT6 | 3'UTR |  |
| IL10RA | cg22324103 | -9.11 | 1.18 | 2.21E-06 | 11 | OPCML | Body |  |
| IL10RA | cg01149712 | -10.24 | 1.34 | 3.51E-06 | 2 | TTC21B | Body | N_Shelf |
| IL10RA | cg07968520 | -7.78 | 1.02 | 3.81E-06 | 1 | PADI4 | Body |  |
| IL10RA | cg26581261 | -9.36 | 1.24 | 5.13E-06 | 8 | ASAP1 | Body; |  |
| IL10RA | cg05906698 | -6.34 | 0.85 | 7.60E-06 | 9 | LINC00583 | Body |  |
| IL10RA | cg03035126 | -8.4 | 1.13 | 7.93E-06 | 9 | ASTN2 | Body; |  |
| IL10RA | cg18832348 | -10 | 1.35 | 1.01E-05 | 5 | TTC37 | Body |  |
| IL10RA | cg27399753 | -9.63 | 1.31 | 1.55E-05 | 16 | CCDC78 | 3'UTR | S_Shore |
| IL10RA | cg19300414 | 24.04 | 3.29 | 1.68E-05 | 2 | PXDN | Body | N_Shore |
| IL10RA | cg14711145 | -9.3 | 1.27 | 1.68E-05 | 5 | UBE2B | Body | S_Shelf |
| IL10RA | cg23188579 | 5.41 | 0.74 | 1.89E-05 | 4 | SCOC | TSS200 |  |
| IL10RA | cg09679693 | -9.79 | 1.35 | 2.33E-05 | 14 | HEATR4 | 5'UTR |  |
| IL10RA | cg08337513 | -10.89 | 1.53 | 4.26E-05 | 6 | CEP85L | Body; |  |
| IL10RA | cg11247803 | -9.08 | 1.28 | 5.87E-05 | 2 | AFF3 | Body; |  |
| IL10RA | cg14891130 | -8.94 | 1.27 | 7.10E-05 | 21 |  |  |  |
| IL10RA | cg00946942 | -11.15 | 1.59 | 7.85E-05 | 5 | CDKL3 | TSS1500; | S_Shore |
| IL10RA | cg00230715 | -4.16 | 0.59 | 8.44E-05 | 5 | EFNA5 | Body |  |
| IL10RA | cg12697199 | -7.65 | 1.12 | 1.93E-04 | 5 |  |  |  |
| IL10RA | cg01027010 | -8.48 | 1.24 | 1.93E-04 | 16 |  |  | N_Shelf |
| IL10RA | cg23513453 | -8.57 | 1.29 | 5.10E-04 | 10 |  |  |  |
| IL10RA | cg10449536 | 15.16 | 2.3 | 7.06E-04 | 18 | KCTD1 | 5'UTR |  |
| IL10RA | cg13236409 | -25.85 | 3.98 | 1.12E-03 | 12 |  |  |  |
| IL10RA | cg07362943 | -8.75 | 1.36 | 1.60E-03 | 1 | ERMAP | 3'UTR; | N_Shelf |
| IL10RA | cg17436889 | -23.1 | 3.6 | 1.63E-03 | 18 | NEDD4L | 5'UTR |  |
| IL10RA | cg06850558 | 9.38 | 1.47 | 1.92E-03 | 3 | DNAH12 | TSS200; |  |
| IL10RA | cg17617387 | 21.2 | 3.38 | 3.15E-03 | 9 | CIZ1 | 5'UTR | N_Shore |
| IL10RA | cg08425114 | -7.94 | 1.27 | 3.21E-03 | 16 |  |  |  |
| IL10RA | cg18623118 | 29.12 | 4.66 | 3.49E-03 | 1 | ILF2 | TSS1500; | S_Shore |
| IL10RA | cg05057292 | 10.74 | 1.74 | 5.33E-03 | 9 |  |  | S_Shelf |
| IL10RA | cg15276051 | -6.32 | 1.03 | 6.49E-03 | 20 | SMOX | Body; |  |
| IL10RA | cg07734293 | -7.4 | 1.22 | 7.67E-03 | 16 |  |  | N_Shelf |
| IL10RA | cg25388939 | -6.64 | 1.09 | 7.67E-03 | 7 | LOC646762 | Body | Island |
| IL10RA | cg18920994 | -7.91 | 1.3 | 7.67E-03 | 4 | SULT1B1 | Body |  |
| IL10RA | cg11904985 | -8.9 | 1.47 | 7.67E-03 | 2 | EDAR | 5'UTR |  |
| IL10RA | cg02294872 | -21.52 | 3.55 | 7.86E-03 | 9 | TEK | Body; |  |
| IL10RA | cg12594793 | -3.74 | 0.62 | 8.80E-03 | 20 |  |  | S_Shelf |
| IL10RA | cg05801066 | -6.87 | 1.14 | 9.23E-03 | 3 | MIR1280 | TSS1500 |  |
| IL10RA | cg10643493 | -22.91 | 3.81 | 1.02E-02 | 8 |  |  |  |
| IL10RA | cg02967752 | -6.8 | 1.14 | 1.06E-02 | 16 |  |  |  |
| IL10RA | cg09758842 | -3.08 | 0.51 | 1.12E-02 | 2 | NCRNA00164 | Body | Island |
| IL10RA | cg01336696 | -6.29 | 1.05 | 1.14E-02 | 3 | SRGAP3 | Body; |  |
| IL10RA | cg00523384 | -6.87 | 1.15 | 1.19E-02 | 4 |  |  |  |
| IL10RA | cg17347650 | -3.68 | 0.62 | 1.26E-02 | 17 | AXIN2 | 3'UTR |  |
| IL10RA | cg00527304 | -7.28 | 1.23 | 1.33E-02 | 3 |  |  |  |
| IL10RA | cg21637884 | 5.91 | 1 | 1.33E-02 | 3 | DNAH12 | 5'UTR |  |
| IL10RA | cg09916698 | 5.03 | 0.85 | 1.37E-02 | 1 | JMJD4 | TSS1500 | Island |
| IL10RA | cg06732450 | -3.69 | 0.62 | 1.37E-02 | 10 | INPP5A | Body |  |
| IL10RA | cg12462429 | -7.11 | 1.2 | 1.37E-02 | 4 | SLC10A6 | Body |  |
| IL10RA | cg25060988 | 8.19 | 1.39 | 1.39E-02 | 11 | CAT | TSS1500 | N_Shore |
| IL10RA | cg15166014 | -7.8 | 1.32 | 1.53E-02 | 1 | KIF1B | Body |  |
| IL10RA | cg22236485 | -7.07 | 1.2 | 1.55E-02 | 8 |  |  | N_Shelf |
| IL10RA | cg24892139 | 42.73 | 7.23 | 1.56E-02 | 5 | SPEF2 | Body; | S_Shore |
| IL10RA | cg11051295 | -3.71 | 0.63 | 1.72E-02 | 4 |  |  | S_Shelf |
| IL10RA | cg21423556 | -12.28 | 2.1 | 1.77E-02 | 11 | ARNTL | Body; |  |
| IL10RA | cg00357578 | -6.62 | 1.14 | 2.13E-02 | 7 |  |  | N_Shelf |
| IL10RA | cg07623363 | -6.32 | 1.09 | 2.15E-02 | 10 |  |  |  |
| IL10RA | cg09249084 | -6.93 | 1.2 | 2.49E-02 | 1 | GPR177 | Body; |  |
| IL10RA | cg18026754 | -6.24 | 1.08 | 2.60E-02 | 22 | SYN3 | Body; |  |
| IL10RA | cg17011132 | 8.61 | 1.5 | 2.90E-02 | 3 | DNAH12 | TSS200; |  |
| IL10RA | cg14820788 | -6.92 | 1.21 | 2.98E-02 | 22 | EIF4ENIF1 | Body; |  |
| IL10RA | cg02185007 | -3.3 | 0.58 | 3.08E-02 | 8 |  |  |  |
| IL10RA | cg10899626 | 18.11 | 3.17 | 3.08E-02 | 14 |  |  |  |
| IL10RA | cg17871757 | -6.52 | 1.14 | 3.35E-02 | 3 | BRPF1 | Body; |  |
| IL10RA | cg22825556 | -6.32 | 1.11 | 3.46E-02 | 6 |  |  |  |
| IL10RA | cg11996434 | 42.63 | 7.5 | 3.70E-02 | 8 | SLCO5A1 | TSS200 | Island |
| IL10RA | cg11815980 | -31.14 | 5.52 | 4.39E-02 | 3 | FHIT | Body; |  |
| IL10RA | cg19761267 | -5.18 | 0.92 | 4.52E-02 | 5 | RAI14 | 5'UTR |  |
| IL10RA | cg22365137 | -7.07 | 1.26 | 4.52E-02 | 12 |  |  |  |
| IL10RA | cg11338576 | -6.82 | 1.21 | 4.54E-02 | 8 |  |  |  |
| NTF3 | cg21913681 | 2.59 | 0.37 | 2.02E-03 | 12 | TBX3 | TSS200; | Island |
| NTF3 | cg06176930 | -2.85 | 0.41 | 2.02E-03 | 1 | MEGF6 | Body |  |
| NTF3 | cg11139878 | -4.13 | 0.6 | 2.02E-03 | 1 | CD5L | TSS200 |  |
| NTF3 | cg08184586 | 12.61 | 1.88 | 2.14E-03 | 19 | CSNK1G2 | TSS1500 | Island |
| NTF3 | cg25024515 | -4.28 | 0.64 | 2.14E-03 | 12 |  |  |  |
| NTF3 | cg14611745 | 2.72 | 0.41 | 2.14E-03 | 1 | LOC100130093 | Body; | N_Shore |
| NTF3 | cg07239814 | -4.18 | 0.63 | 2.14E-03 | 5 |  |  |  |
| NTF3 | cg19356748 | -4.05 | 0.61 | 2.14E-03 | 2 | TTN | Body; |  |
| NTF3 | cg12390081 | -2.13 | 0.32 | 2.14E-03 | 6 | EXOC2 | 5'UTR |  |
| NTF3 | cg14339216 | 4.91 | 0.74 | 2.14E-03 | 19 | DHX34 | 5'UTR | S_Shore |
| NTF3 | cg06900861 | 12.81 | 1.93 | 2.14E-03 | 8 | LRRCC1 | TSS200; | N_Shore |
| NTF3 | cg05304979 | 2.28 | 0.34 | 2.14E-03 | 16 | LOC100129637 | Body | N_Shore |
| NTF3 | cg16624824 | -4.29 | 0.65 | 2.14E-03 | 4 | CCDC158 | Body |  |
| NTF3 | cg27348370 | -3.88 | 0.59 | 2.14E-03 | 7 | LMOD2 | TSS1500 |  |
| NTF3 | cg14496016 | 2.63 | 0.4 | 2.14E-03 | 1 | PCP4L1 | TSS200 | Island |
| NTF3 | cg27427357 | 2.85 | 0.43 | 2.14E-03 | 6 | HIST1H4B | TSS200 | Island |
| NTF3 | cg03965044 | 2.24 | 0.34 | 2.14E-03 | 17 | ABCC3 | TSS200; | Island |
| NTF3 | cg25730670 | -2.15 | 0.33 | 2.14E-03 | 7 | LEP | 5'UTR |  |
| NTF3 | cg09195920 | 5.67 | 0.87 | 2.14E-03 | 14 | MPP5 | TSS1500 | N_Shore |
| NTF3 | cg17307742 | -4.25 | 0.65 | 2.14E-03 | 1 | CD58 | Body; |  |
| NTF3 | cg08397205 | -1.94 | 0.3 | 2.43E-03 | 22 |  |  | S_Shelf |
| NTF3 | cg11086737 | -4.17 | 0.64 | 2.56E-03 | 15 | TMCO5B | Body |  |
| NTF3 | cg02593579 | 10.55 | 1.63 | 2.60E-03 | 12 | C12orf49 | 5'UTR | Island |
| NTF3 | cg01296875 | 2.66 | 0.41 | 2.60E-03 | 3 | ALG3 | TSS200 | N_Shore |
| NTF3 | cg15727320 | -4.37 | 0.68 | 3.94E-03 | 12 | P11 | Body |  |
| NTF3 | cg00737548 | -1.99 | 0.31 | 4.39E-03 | 9 | INSL6 | Body |  |
| NTF3 | cg21796322 | -4.39 | 0.69 | 4.44E-03 | 3 | C3orf21 | Body |  |
| NTF3 | cg16819685 | -4.05 | 0.64 | 4.44E-03 | 6 |  |  |  |
| NTF3 | cg07777609 | -4.14 | 0.65 | 4.44E-03 | 15 | IDH3A | Body |  |
| NTF3 | cg02433545 | -4.23 | 0.67 | 4.44E-03 | 15 | RORA | Body | N_Shelf |
| NTF3 | cg14683750 | -2.07 | 0.33 | 4.44E-03 | 15 | TMEM62 | Body |  |
| NTF3 | cg02334098 | -2.08 | 0.33 | 4.44E-03 | 12 |  |  |  |
| NTF3 | cg18138031 | -2.05 | 0.32 | 4.53E-03 | 3 | ATXN7 | 5'UTR | N_Shore |
| NTF3 | cg22985036 | -1.93 | 0.31 | 4.62E-03 | 5 | POLS | Body |  |
| NTF3 | cg06480070 | -4.29 | 0.69 | 5.52E-03 | 15 | CEMIP | Body; |  |
| NTF3 | cg23689697 | -2.04 | 0.33 | 5.56E-03 | 2 | SSB | Body |  |
| NTF3 | cg22146312 | 6.68 | 1.07 | 5.67E-03 | 19 | DHX34 | 5'UTR | Island |
| NTF3 | cg24689976 | -2.01 | 0.32 | 5.68E-03 | 6 | SLC35A1 | Body; | S_Shelf |
| NTF3 | cg02182074 | -4 | 0.64 | 6.21E-03 | 20 | FERMT1 | TSS1500 | S_Shore |
| NTF3 | cg23024108 | -3.95 | 0.64 | 6.24E-03 | 4 |  |  |  |
| NTF3 | cg20353344 | -2.07 | 0.33 | 6.71E-03 | 11 | RNASEH2C | TSS1500 | S_Shore |
| NTF3 | cg18410551 | -3.9 | 0.63 | 7.82E-03 | 1 | KDM4A | 3'UTR | N_Shelf |
| NTF3 | cg07913096 | 8.54 | 1.39 | 8.45E-03 | 1 |  |  | Island |
| NTF3 | cg22249386 | -5.5 | 0.9 | 8.83E-03 | 17 | MYH13 | Body |  |
| NTF3 | cg12798657 | -4.01 | 0.66 | 8.83E-03 | 17 | RAP1GAP2 | Body; |  |
| NTF3 | cg13048896 | -4.7 | 0.77 | 9.37E-03 | 16 |  |  |  |
| NTF3 | cg10745724 | -3.9 | 0.64 | 9.76E-03 | 3 | CCDC12 | Body; |  |
| NTF3 | cg00688487 | -3.97 | 0.65 | 1.05E-02 | 1 | SMYD3 | Body; |  |
| NTF3 | cg27059530 | 11.72 | 1.94 | 1.17E-02 | 14 | MPP5 | TSS1500 | N_Shore |
| NTF3 | cg08915683 | -5.02 | 0.83 | 1.17E-02 | 16 |  |  |  |
| NTF3 | cg19181479 | -2.06 | 0.34 | 1.22E-02 | 10 | ZNF488 | 3'UTR | S_Shelf |
| NTF3 | cg24757997 | -2.19 | 0.37 | 1.43E-02 | 4 |  |  |  |
| NTF3 | cg15640734 | -9.01 | 1.51 | 1.47E-02 | 5 | SLC9A3 | Body | Island |
| NTF3 | cg18869444 | -9.8 | 1.65 | 1.56E-02 | 2 |  |  |  |
| NTF3 | cg14217074 | -3.81 | 0.64 | 1.56E-02 | 3 |  |  |  |
| NTF3 | cg05359280 | -7.49 | 1.26 | 1.58E-02 | 4 | ZFP42 | TSS1500; | N_Shore |
| NTF3 | cg10634702 | -5.78 | 0.97 | 1.63E-02 | 5 | SLC9A3 | Body | Island |
| NTF3 | cg20003494 | 2.25 | 0.38 | 1.63E-02 | 4 | SNCA | 5'UTR | N_Shore |
| NTF3 | cg06961160 | -4.18 | 0.7 | 1.63E-02 | 21 | RCAN1 | TSS1500 |  |
| NTF3 | cg03056087 | -1.93 | 0.33 | 1.63E-02 | 14 | PPP1R13B | Body |  |
| NTF3 | cg12232901 | -3.94 | 0.66 | 1.65E-02 | 1 | LGR6 | Body; |  |
| NTF3 | cg15671158 | 6 | 1.01 | 1.76E-02 | 5 | CAMLG | TSS1500 | N_Shore |
| NTF3 | cg09001112 | -5.75 | 0.98 | 1.76E-02 | 17 | ITGB4 | Body; | Island |
| NTF3 | cg17419619 | -2.88 | 0.49 | 2.20E-02 | 7 | CUX1 | Body; |  |
| NTF3 | cg03475429 | -5.21 | 0.89 | 2.20E-02 | 10 |  |  |  |
| NTF3 | cg15151778 | -1.92 | 0.33 | 2.59E-02 | 8 | BOP1 | Body | Island |
| NTF3 | cg12554944 | -1.93 | 0.33 | 2.59E-02 | 8 | GTF2E2 | Body |  |
| NTF3 | cg07148744 | 6.9 | 1.19 | 2.60E-02 | 1 |  |  | Island |
| NTF3 | cg04650789 | 5.57 | 0.96 | 2.78E-02 | 15 |  |  | N_Shore |
| NTF3 | cg05803265 | -4.48 | 0.77 | 2.98E-02 | 1 | YIPF1 | Body | N_Shelf |
| NTF3 | cg17202331 | -3.64 | 0.63 | 2.98E-02 | 20 | BTBD3 | TSS1500 |  |
| NTF3 | cg15645815 | -3.59 | 0.63 | 4.17E-02 | 21 |  |  |  |
| NTF3 | cg25774237 | 16.06 | 2.82 | 4.17E-02 | 8 | LRRCC1 | TSS1500 | N_Shore |
| NTF3 | cg04362858 | 4.26 | 0.75 | 4.34E-02 | 3 |  |  | Island |
| NTF3 | cg04658858 | -2.96 | 0.52 | 4.59E-02 | 22 | TTC28-AS1 | Body |  |
| NTF3 | cg15230717 | -6.17 | 1.09 | 4.64E-02 | 20 |  |  |  |
| NTF3 | cg06981033 | -3.39 | 0.6 | 4.85E-02 | 16 |  |  |  |
| TNFRSF9 | cg24468104 | -5.71 | 0.8 | 1.43E-03 | 7 | LINC01006 | Body |  |
| TNFRSF9 | cg21184859 | 2.99 | 0.43 | 3.30E-03 | 12 | PTHLH | TSS200 | Island |
| TNFRSF9 | cg08000459 | -2.71 | 0.4 | 3.55E-03 | 4 |  |  | S_Shore |
| TNFRSF9 | cg00222053 | -5.34 | 0.8 | 3.55E-03 | 20 | GHRH | TSS1500 |  |
| TNFRSF9 | cg16893634 | -5.1 | 0.76 | 3.55E-03 | 3 |  |  | N_Shelf |
| TNFRSF9 | cg06827632 | -2.66 | 0.4 | 3.55E-03 | 12 | TSPAN11 | Body |  |
| TNFRSF9 | cg14078101 | 2.85 | 0.43 | 3.55E-03 | 9 | UHRF2 | TSS1500 | Island |
| TNFRSF9 | cg05764704 | -5.87 | 0.89 | 3.55E-03 | 6 | SLC35A1 | Body; |  |
| TNFRSF9 | cg09140833 | -2.6 | 0.4 | 4.31E-03 | 14 | CHD8 | TSS1500 |  |
| TNFRSF9 | cg26404919 | -5.74 | 0.88 | 4.31E-03 | 19 | SMARCA4 | Body; |  |
| TNFRSF9 | cg13152584 | -4.65 | 0.72 | 4.87E-03 | 18 | PIEZO2 | Body |  |
| TNFRSF9 | cg21233897 | -2.7 | 0.42 | 4.87E-03 | 6 | TFEB | Body; |  |
| TNFRSF9 | cg17721480 | -4.79 | 0.75 | 5.38E-03 | 4 | FAT1 | Body |  |
| TNFRSF9 | cg06172310 | -5.29 | 0.82 | 5.38E-03 | 8 | MTDH | Body |  |
| TNFRSF9 | cg05997278 | -4.89 | 0.76 | 5.64E-03 | 2 | ANTXR1 | Body; |  |
| TNFRSF9 | cg03813291 | -2.51 | 0.39 | 6.17E-03 | 12 | SLC11A2 | ExonBnd |  |
| TNFRSF9 | cg02873048 | -5.14 | 0.81 | 6.17E-03 | 2 |  |  |  |
| TNFRSF9 | cg12039910 | -4.85 | 0.76 | 6.17E-03 | 15 | NIPA1 | Body; |  |
| TNFRSF9 | cg08478006 | -5.01 | 0.79 | 6.17E-03 | 16 |  |  | N_Shore |
| TNFRSF9 | cg19756821 | 2.81 | 0.44 | 6.17E-03 | 1 |  |  | Island |
| TNFRSF9 | cg04092513 | 4.82 | 0.76 | 6.17E-03 | 2 | C2orf60 | TSS200 | N_Shore |
| TNFRSF9 | cg18181145 | -5.42 | 0.86 | 6.25E-03 | 7 | CRYGN | 3'UTR | N_Shelf |
| TNFRSF9 | cg05170705 | -4.47 | 0.71 | 6.25E-03 | 17 | NEURL4 | ExonBnd | S_Shelf |
| TNFRSF9 | cg08508527 | -5.06 | 0.81 | 7.86E-03 | 2 | RPL31 | Body; | S_Shelf |
| TNFRSF9 | cg14391441 | -5.16 | 0.83 | 8.51E-03 | 8 | COLEC10 | Body |  |
| TNFRSF9 | cg18930905 | -2.58 | 0.42 | 8.62E-03 | 4 | KIAA1211 | Body | Island |
| TNFRSF9 | cg15871214 | 2.78 | 0.45 | 1.05E-02 | 7 | MRPL32 | TSS1500 | N_Shore |
| TNFRSF9 | cg14457998 | -5.46 | 0.89 | 1.38E-02 | 10 |  |  |  |
| TNFRSF9 | cg17930169 | -5.11 | 0.84 | 1.62E-02 | 14 | SNORD113-7 | TSS1500 |  |
| TNFRSF9 | cg15791105 | -3.13 | 0.52 | 1.66E-02 | 12 | COL2A1 | 3'UTR; |  |
| TNFRSF9 | cg16324767 | -4.26 | 0.7 | 1.66E-02 | 10 | MIR1296 | TSS200 |  |
| TNFRSF9 | cg01513623 | -2.55 | 0.42 | 1.75E-02 | 16 | GPRC5B | Body; |  |
| TNFRSF9 | cg10869730 | -2.4 | 0.4 | 2.42E-02 | 14 |  |  |  |
| TNFRSF9 | cg20998686 | -4.69 | 0.79 | 2.42E-02 | 14 |  |  |  |
| PLAU | cg17168630 | -3.77 | 0.42 | 3.45E-08 | 19 | PLAUR | TSS1500 | S_Shore |
| CD244 | cg07240937 | 11.05 | 1.67 | 2.69E-02 | 1 | NPR1 | TSS200 | Island |
