# Supplementary material for: Integrative analysis of DNA methylation and inflammatory protein biomarkers in hypertension
Source: Front Immunol. 2026 Feb 11;17:1671540. doi: 10.3389/fimmu.2026.1671540 (PMC12932573; doi:10.3389/fimmu.2026.1671540)
Supplement: Supplementary file 5 [file Table3.docx]

Supplementary table 3. Association of the methylation levels of pQTMs with CIMT

| CpG | cor | FDR | ref Gene | related Protein |
| --- | --- | --- | --- | --- |
| cg18652997 | -1.709 | 2.12E-03 | SORCS2 | CD274 |
| cg10640435 | 8.840 | 2.19E-03 | HAPLN2 | FGF23 |
| cg02805410 | -8.855 | 1.97E-04 | MIR1273E | FGF23 |
| cg05611414 | -2.522 | 1.61E-03 | SNAR-D | FGF23 |
| cg11364888 | -5.944 | 4.93E-03 |  | FGF23 |
| cg03965044 | 5.640 | 4.81E-13 | ABCC3 | NTF3 |
| cg01296875 | 19.303 | 5.56E-26 | ALG3 | NTF3 |
| cg18138031 | -5.192 | 3.56E-45 | ATXN7 | NTF3 |
| cg15151778 | -7.195 | 5.36E-28 | BOP1 | NTF3 |
| cg17202331 | -5.321 | 1.03E-05 | BTBD3 | NTF3 |
| cg21796322 | -20.030 | 8.94E-12 | C3orf21 | NTF3 |
| cg15671158 | 14.435 | 2.68E-06 | CAMLG | NTF3 |
| cg10745724 | -2.844 | 2.11E-02 | CCDC12 | NTF3 |
| cg16624824 | -8.885 | 4.46E-06 | CCDC158 | NTF3 |
| cg17307742 | -18.600 | 1.07E-08 | CD58 | NTF3 |
| cg11139878 | -12.700 | 9.96E-09 | CD5L | NTF3 |
| cg06480070 | -20.758 | 5.51E-09 | CEMIP | NTF3 |
| cg08184586 | 10.489 | 1.66E-03 | CSNK1G2 | NTF3 |
| cg17419619 | -12.760 | 1.85E-08 | CUX1 | NTF3 |
| cg14339216 | 25.800 | 3.29E-08 | DHX34 | NTF3 |
| cg22146312 | 19.029 | 2.56E-07 | DHX34 | NTF3 |
| cg12390081 | -8.939 | 8.70E-69 | EXOC2 | NTF3 |
| cg02182074 | -23.158 | 5.02E-14 | FERMT1 | NTF3 |
| cg12554944 | -5.913 | 6.24E-54 | GTF2E2 | NTF3 |
| cg27427357 | 22.074 | 7.08E-12 | HIST1H4B | NTF3 |
| cg07777609 | -14.931 | 1.18E-08 | IDH3A | NTF3 |
| cg00737548 | -3.455 | 2.54E-26 | INSL6 | NTF3 |
| cg09001112 | -4.286 | 5.23E-03 | ITGB4 | NTF3 |
| cg18410551 | -14.375 | 4.79E-06 | KDM4A | NTF3 |
| cg25730670 | -8.845 | 2.28E-54 | LEP | NTF3 |
| cg12232901 | -5.111 | 3.69E-04 | LGR6 | NTF3 |
| cg27348370 | -2.736 | 1.53E-03 | LMOD2 | NTF3 |
| cg05304979 | 6.694 | 7.86E-18 | LOC100129637 | NTF3 |
| cg14611745 | 10.206 | 1.18E-06 | LOC100130093 | NTF3 |
| cg06900861 | 21.495 | 3.36E-13 | LRRCC1 | NTF3 |
| cg25774237 | 26.537 | 2.52E-04 | LRRCC1 | NTF3 |
| cg06176930 | -3.394 | 4.99E-07 | MEGF6 | NTF3 |
| cg09195920 | 20.932 | 2.10E-08 | MPP5 | NTF3 |
| cg27059530 | 52.040 | 7.20E-12 | MPP5 | NTF3 |
| cg22249386 | -5.368 | 1.20E-04 | MYH13 | NTF3 |
| cg15727320 | -16.850 | 1.01E-09 | P11 | NTF3 |
| cg14496016 | 17.709 | 3.15E-12 | PCP4L1 | NTF3 |
| cg22985036 | -4.022 | 4.33E-31 | POLS | NTF3 |
| cg03056087 | -5.054 | 2.58E-41 | PPP1R13B | NTF3 |
| cg12798657 | -8.251 | 4.69E-05 | RAP1GAP2 | NTF3 |
| cg06961160 | -13.788 | 1.72E-08 | RCAN1 | NTF3 |
| cg20353344 | -7.434 | 1.02E-61 | RNASEH2C | NTF3 |
| cg02593579 | 27.617 | 6.18E-10 | RNFT2 | NTF3 |
| cg02433545 | -7.471 | 6.06E-06 | RORA | NTF3 |
| cg24689976 | -6.294 | 1.07E-47 | SLC35A1 | NTF3 |
| cg10634702 | -9.407 | 5.99E-04 | SLC9A3 | NTF3 |
| cg15640734 | -20.174 | 2.81E-05 | SLC9A3 | NTF3 |
| cg00688487 | -13.600 | 1.74E-07 | SMYD3 | NTF3 |
| cg20003494 | 7.505 | 7.62E-27 | SNCA | NTF3 |
| cg23689697 | -5.497 | 1.97E-40 | SSB | NTF3 |
| cg21913681 | 8.792 | 6.66E-28 | TBX3;TBX3 | NTF3 |
| cg11086737 | -8.215 | 3.17E-05 | TMCO5B | NTF3 |
| cg14683750 | -6.924 | 5.35E-61 | TMEM62 | NTF3 |
| cg04658858 | -9.289 | 4.00E-07 | TTC28-AS1 | NTF3 |
| cg19356748 | -18.795 | 3.36E-08 | TTN | NTF3 |
| cg05803265 | -6.910 | 4.89E-05 | YIPF1 | NTF3 |
| cg05359280 | -16.357 | 1.13E-06 | ZFP42 | NTF3 |
| cg19181479 | -5.854 | 2.16E-39 | ZNF488 | NTF3 |
| cg02334098 | -8.473 | 2.86E-66 |  | NTF3 |
| cg04362858 | 3.918 | 1.16E-03 |  | NTF3 |
| cg04650789 | 9.360 | 5.14E-05 |  | NTF3 |
| cg06981033 | -6.196 | 2.17E-04 |  | NTF3 |
| cg07148744 | 25.868 | 5.05E-08 |  | NTF3 |
| cg07239814 | -5.331 | 4.21E-05 |  | NTF3 |
| cg07913096 | 29.932 | 2.57E-07 |  | NTF3 |
| cg08397205 | -3.290 | 4.36E-25 |  | NTF3 |
| cg08915683 | -8.791 | 5.39E-06 |  | NTF3 |
| cg13048896 | -17.735 | 1.92E-07 |  | NTF3 |
| cg14217074 | -9.519 | 1.13E-06 |  | NTF3 |
| cg15230717 | -9.829 | 4.99E-06 |  | NTF3 |
| cg15645815 | -7.904 | 1.11E-06 |  | NTF3 |
| cg16819685 | -11.808 | 3.02E-07 |  | NTF3 |
| cg18869444 | -13.278 | 4.54E-04 |  | NTF3 |
| cg23024108 | -27.565 | 2.20E-09 |  | NTF3 |
| cg24757997 | -3.518 | 6.30E-07 |  | NTF3 |
| cg25024515 | -31.310 | 2.17E-15 |  | NTF3 |
